# Glutathione S-Transferase Gene Family in *Gossypium raimondii* and *G. arboreum*: Comparative Genomic Study and their Expression under Salt Stress

**DOI:** 10.3389/fpls.2016.00139

**Published:** 2016-02-12

**Authors:** Yating Dong, Cong Li, Yi Zhang, Qiuling He, Muhammad K. Daud, Jinhong Chen, Shuijin Zhu

**Affiliations:** ^1^Department of Agronomy, Zhejiang UniversityHangzhou, China; ^2^Department of Biotechnology and Genetic Engineering, Kohat University of Science and TechnologyKohat, Pakistan

**Keywords:** salt stress, cotton, phylogenetic analysis, gene family, GST

## Abstract

Glutathione S-transferases (GSTs) play versatile functions in multiple aspects of plant growth and development. A comprehensive genome-wide survey of this gene family in the genomes of *G. raimondii* and *G. arboreum* was carried out in this study. Based on phylogenetic analyses, the *GST* gene family of both two diploid cotton species could be divided into eight classes, and approximately all the *GST* genes within the same subfamily shared similar gene structure. Additionally, the gene structures between the orthologs were highly conserved. The chromosomal localization analyses revealed that *GST* genes were unevenly distributed across the genome in both *G. raimondii* and *G. arboreum*. Tandem duplication could be the major driver for the expansion of *GST* gene families. Meanwhile, the expression analysis for the selected 40 *GST* genes showed that they exhibited tissue-specific expression patterns and their expression were induced or repressed by salt stress. Those findings shed lights on the function and evolution of the *GST* gene family in *Gossypium* species.

## Introduction

Glutathione S-transferases (GSTs; EC 2.5.1.18) are ancient and ubiquitous proteins encoded by a large gene family that function versatilely in organism. As a kind of detoxification enzymes, GSTs catalyze the conjugation of the tripeptide glutathione (GSH) to a variety of hydrophobic, electrophilic, and usually cytotoxic exogenous compounds (Marrs, 1996). There are cytosolic, mitochondrial and microsomal GSTs derived from a gene superfamily that are involved in the metabolism of xenobiotics (Armstrong, 1997). In general, microsomal and mitochondrial GSTs show great differences in biosynthesis and sequence identity with cytosolic GSTs (Mohsenzadeh et al., 2011). In plants, most cytosolic GSTs typically function as either heterodimer or homodimer of subunits ranging from 23 to 29 kDa in molecular weight (Frova, 2006). Each subunit contains a conserved GSH-binding site (G-site) in the N-terminal domain and an electrophilic substrate binding site (H-site) located in the C-terminal domain (Edwards et al., 2000). GSTs can also be monomeric, like DHAR and Lambda GST in *Arabidopsis* (Dixon et al., 2002). GSTs comprise ~2% of soluble proteins in plants (Rezaei et al., 2013). Based on gene organization and amino acid sequence similarity, the soluble GSTs can be divided into eight classes, including Phi (F), Tau (U), Lambda (L), dehydroascorbate reductase (DHAR), Theta (T), Zeta (Z), γ-subunit of translation elongation factor 1B (EF1Bγ), and tetrachlorohydroquinone dehalogenase (TCHQD; Sheehan et al., 2001; Dixon et al., 2002; Liu et al., 2013). Among these, the first four classes are specific to plant. Genome-wide analyses have indicated that there were 55 *GST* genes in *Arabidopsis* (Sappl et al., 2009), 79 in rice (Soranzo et al., 2004; Jain et al., 2010), 84 in barley (Rezaei et al., 2013), 23 in sweet orange (Licciardello et al., 2014), and 27 in Japanese larch (Yang et al., 2014).

Since the function for plant GSTs in herbicides detoxification was firstly detected, many researches have focused on their functions under various stimulations. It has been confirmed that GSTs can be induced by plant hormones such as auxins, ABA, and ethylene, as well as biotic and abiotic stresses (Dixon et al., 1998). To date, abundant *GST* genes have been characterized from numerous plant species. Among these *GST* genes, Tau and Phi classes are most investigated probably because of their abundant presence in plant kingdom. *AtGSTU26* in *Arabidopsis* was induced by the chloroacetanilide herbicides, alachlor and metolachlor, the safener benoxacor, and low temperatures (Nutricati et al., 2006). The *OsGSTU5* in rice shown high activity toward chloro-s-triazine and acetanilide herbicides (Cho et al., 2006), and overexpression of *OsGSTU4* in *Arabidopsis* improved the tolerance to salinity and oxidative stresses (Sharma et al., 2014). The expression levels of *TaGSTU1B* and *TaGSTF6* were increased under drought stress in wheat (Galle et al., 2009). Meanwhile, 35 of 56 *SbGSTUs* in Sorghum shown significant response to abiotic stresses including cold, PEG and high salinity (Chi et al., 2011). The expression of *GmGSTL1* from soybean in transgenic *Arabidopsis* could also alleviate the symptoms under salt stress (Chan and Lam, 2014). Many other similar researches on GST family and their functions were reported recently (Urano et al., 2000; Thom et al., 2001; Ma et al., 2009; Ji et al., 2010). However, little is known about this gene family in cotton, especially their function under salt stress.

Cotton, which belongs to the genus of *Gossypium*, is considered the main source of natural fiber and cultivated worldwide. There are ~45 diploid (2*n* = 2*x* = 26) and 5 tetraploid (2*n* = 4*x* = 52) species. Cotton is an ideal model system for plant polyploid research (Kadir, 1976; Grover et al., 2007). With completion of the genome sequencing of the two diploid cotton species, *G. raimondii* (Paterson et al., 2012; Wang et al., 2012) and *G. arboreum* (Li et al., 2014), genome-wide analyses of all related genes have been realized. *G. raimondii* and *G. arboreum* were the putative donor species for the D and A chromosome groups of tetraploid cotton species, respectively (Kadir, 1976; Grover et al., 2007). Here, we conducted a systematic study of *GST* gene family in *G. raimondii* and *G. arboreum* to identify the characterization and phylogenetic relationships between the two species. Functional diversification and expression profiles of *GST* genes in response to salt stress were also investigated. It may elucidate the evolution mechanism of *GST* gene family in cotton, which will also promote us to perform a further investigation on the stress responsive genes that will provide valuable information for breeding stress-resistant cotton.

## Materials and methods

### Sequence retrial and annotation of *GST* genes

The *G. raimondii* genome database (release version 2.1; Paterson et al., 2012) was obtained from Phytozome (http://phytozome.jgi.doe.gov/pz/portal.html#!info?alias=Org_Graimondii). The published GST proteins of *Arabidopsis* (Sappl et al., 2009) and rice (Soranzo et al., 2004; Jain et al., 2010) were downloaded from the Arabidopsis Information Resource (TAIR release 10, http://www.arabidopsis.org) and the Rice Genome Annotation Project Database (RGAP release 7, http://rice.plantbiology.msu.edu/index.shtml), respectively. Afterwards, they were used as queries in BlastP and tBlastN searches with a stringent *E*-value cut-off (≤ e−20) against the *G. raimondii* genome database. Then, all significant hits were subjected to the InterProScan program (http://www.ebi.ac.uk/Tools/pfa/iprscan/; Quevillon et al., 2005) to confirm the presence of the conserved domains. Pfam (http://pfam.sanger.ac.uk/; Finn et al., 2008, 2014) and SMART (http://smart.embl-heidelberg.de/; Letunic et al., 2015) database were applied to further determine each candidate member of the GST family. The same approaches were executed to search against the *G. arboreum* genome database (release version2; Li et al., 2014) which was downloaded from CGP (http://cgp.genomics.org.cn/) to get the putative homologous *GST* genes. Finally, the physicochemical parameters of the full-length proteins were calculated by Compute pI/Mw tool (http://web.expasy.org/compute_pi/pi_tool; Bjellqvist et al., 1994), and the subcellular localization prediction was predicted by the CELLO v2.5 server (http://cello.life.nctu.edu.tw/; Yu et al., 2004).

### Phylogenetic analysis and genomic organizations prediction

Multiple sequence alignments of all full-length GST proteins were performed using MUSCLE 3.52 program (Edgar, 2004) with default parameters, followed by manual comparisons and refinements. Phylogenetic trees were constructed by Neighbor Joining method in MEGA 5.2 (Tamura et al., 2011). Neighbor Joining analyses were carried out using pairwise deletion option and poisson correction model. To assess statistical reliability for each node, bootstrap tests were conducted with 1000 replicates. Furthermore, Minimum Evolution method of MEGA was also applied in the tree construction to validate the results from the NJ method.

The exon-intron structures were deduced using GSDS (http://gsds.cbi.pku.edu.cn/; Hu et al., 2015), through comparing the predicted coding sequences and their corresponding genomic DNA sequences.

### Chromosomal localization and detection of gene duplication

All the cotton *GST* genes were mapped on the chromosomes according to their starting positions given in the genome annotation document. The chromosome location images were portrayed graphically by MapInspect software.

*GST* gene duplication events were defined according to the length coverage of the longer one between aligned gene sequences and the identity of the aligned regions, and only one duplication event was counted for tightly-linked genes (Maher et al., 2006; Ouyang et al., 2009; Liu et al., 2014). Referring to the different chromosomal location, these *GST* genes were designated as either tandem duplication or segmental duplication.

### Estimating Ka/Ks ratios for duplicated gene pairs

Firstly, all the full-length gene sequences of the duplicated *GST* gene pairs of *G. raimondii* and *G. arboreum* were aligned by Clustal X 2.0 program (Larkin et al., 2007). Subsequently, the nonsynonymous substitutions rate (Ka) and synonymous substitution rate (Ks) were calculated using the software DnaSp V5.0 (Librado and Rozas, 2009). Eventually, the selection pressure of each gene pair was assessed based on the Ka/Ks ratio.

### Promoter regions analysis

In order to analyze promoter, the 2500 bp genomic DNA sequences upstream of the initiation codon (ATG) were extracted from the genome database. Then, these sequences were subjected to the PLACE database (http://www.dna.affrc.go.jp/PLACE/signalscan.html; Higo et al., 1999) to search for the putative *cis*-elements in promoter regions.

### Plant materials and salt treatments

One-week-old cotton seedlings of *G. raimondii* and *G. arboreum* were transplanted into polypots (10 cm in diameter) with MS medium and put in a temperature-controlled chamber with temperature of 28°C, relative humidity of 60%, and photoperiod of 16 h light and 8 h dark. After acclimatization for 7 days, they were subjected to salt treatment. For *G. raimondii*, the MS solution were adjusted to desired salt concentrations, i.e., 0, 50, 100, and 200 mM, which represented the control condition, slight stress, moderate stress, and severe stress, respectively. Identically, final salt concentrations for *G. arboreum* were 0, 100, 200, and 300 mM. Three biological replicates were conducted for each sample. After treatments for 2 weeks, the root, stem, cotyledon and leaf were harvested from each individual for expression analysis. All collected samples were immediately frozen in liquid nitrogen and stored at −80°C.

### RNA isolation and real-time quantitative PCR (qRT-PCR) analysis

Total RNAs of all the collected samples were extracted using EASYspin Plus RNAprep Kit (Aidlab, Beijing, China). The quantity and quality were determined by a NanoDrop 2000 Spectrophotometer (NanoDrop Technologies, Wilmington, DE, USA). First-strand cDNA was synthesized with PrimerScript 1st Strand cDNA synthesis kit (TaKaRa, Dalian, China). All the protocols followed to the manufacturer's instructions. qRT-PCR was performed with Lightcycler 96 system (Roche, Mannheim, Germany) using SYBR the premix Ex taq (TakaRa, Dalian, China) in 20 μL volume according to the supplier's protocols. The specific primers used were listed in Supplementary Table 1, and cotton *UBQ7* was used as an internal control. Three biological replicates were performed for each sample. The relative expression levels were calculated according to the 2^−ΔΔCt^ method (Livak and Schmittgen, 2001). The heatmap for expression profiles were generated with the Mev 4.0 software (Saeed et al., 2003).

## Results

### Characterization of *GST* gene family in *G. raimondii* and *G. arboreum*

The genome-wide analyses of *GST* gene family have been performed on the basis of recently completed two diploid cotton genome sequences, *G. raimondii* (Paterson et al., 2012) and *G. arboreum* (Li et al., 2014). Through a systematic BLAST search against the *G. raimondii* and *G. arboreum* genome databases with the query sequences of *Arabidopsis* (55) and rice (77) GST proteins, the candidate *GST* genes were identified. Among the 79 *GST* genes in rice (Soranzo et al., 2004; Jain et al., 2010), the sequences for two genes (LOC_Os10g38501, *OsGSTU3*; LOC_Os10g38495, *OsGSTU4*) could not be retrieved as they have become obsolete entries in TIGR database. Therefore, the number of rice *GST* genes used as queries was 77. Then, all these retrieved sequences were verified by the Pfam and SMART analyses, and a total of 59 and 49 non-redundant genes containing both typical GST N- and C-terminal domains were confirmed in the *G. raimondii* and *G. arboreum* genome, respectively (Tables 1, 2).

**Table 1:** **The information of 59 *GST* genes from *G. raimondii***.

**Gene Name**	**Gene identifier**	**Genomics position**	**CDS**	**Size (AA)**	***Mw(kDa)***	***pI***	**Predicted Subcellular localization**	**Strand**
*GrGSTU1*	Gorai.001G204000.1	Chr01: 39690577-39691331	663	220	25.12	5.66	Cytoplasmic	Plus
*GrGSTU2*	Gorai.002G166900.1	Chr02: 41370934-41371707	684	227	26.02	6.4	Cytoplasmic	Minus
*GrGSTU3*	Gorai.003G127500.1	Chr03: 37860854-37862140	666	221	25.71	6.03	Cytoplasmic	Minus
*GrGSTU4*	Gorai.003G127600.1	Chr03: 37865020-37866250	711	236	27.40	6.61	Cytoplasmic	Minus
*GrGSTU5*	Gorai.004G031900.1	Chr04: 2613632-2615489	723	240	26.84	6.23	Plasma Membrane	Plus
*GrGSTU6*	Gorai.004G032100.1	Chr04: 2626962-2628597	723	240	26.76	6.98	Chloroplast	Plus
*GrGSTU7*	Gorai.005G036300.1	Chr05: 3450978-3452932	744	247	29.10	8.49	Cytoplasmic	Minus
*GrGSTU8*	Gorai.005G037700.1	Chr05: 3540959-3542789	675	224	26.47	6.09	Cytoplasmic	Minus
*GrGSTU9*	Gorai.005G037800.1	Chr05: 3544165-3545392	675	224	26.38	6.09	Cytoplasmic	Minus
*GrGSTU10*	Gorai.005G037900.1	Chr05: 3566839-3573242	672	223	25.73	5.77	Cytoplasmic	Minus
*GrGSTU11*	Gorai.005G038200.1	Chr05: 3598187-3600069	678	225	26.53	6.13	Cytoplasmic	Minus
*GrGSTU12*	Gorai.005G038500.1	Chr05: 3628470-3631039	672	223	25.78	5.42	Cytoplasmic	Minus
*GrGSTU13*	Gorai.005G038700.1	Chr05: 3671704-3675708	669	222	25.84	6.18	Cytoplasmic	Minus
*GrGSTU14*	Gorai.005G038800.1	Chr05: 3683779-3685218	630	209	24.31	7.13	Cytoplasmic	Minus
*GrGSTU15*	Gorai.006G178400.1	Chr06: 43579129-43580293	654	217	24.79	5.26	Cytoplasmic	Plus
*GrGSTU16*	Gorai.006G178600.1	Chr06: 43599284-43600758	678	225	25.85	5.75	Cytoplasmic	Plus
*GrGSTU17*	Gorai.006G178700.1	Chr06: 43603138-43603967	594	197	22.25	5.53	Cytoplasmic	Plus
*GrGSTU18*	Gorai.007G072000.1	Chr07: 5072423-5074861	702	233	26.13	8.67	Cytoplasmic	Plus
*GrGSTU19*	Gorai.007G151400.1	Chr07: 12969311-12970236	672	223	25.82	5.61	Cytoplasmic	Minus
*GrGSTU20*	Gorai.007G245500.1	Chr07: 36874442-36875934	699	232	26.06	6.01	Cytoplasmic	Minus
*GrGSTU21*	Gorai.007G249200.1	Chr07: 39160450-39161476	663	220	25.32	5.52	Cytoplasmic	Plus
*GrGSTU22*	Gorai.007G348100.1	Chr07: 57798231-57800653	627	208	24.65	9.35	Mitochondrial	Minus
*GrGSTU23*	Gorai.009G357800.1	Chr09: 46937960-46939545	666	221	25.79	5.72	Cytoplasmic	Minus
*GrGSTU24*	Gorai.010G115400.1	Chr10: 22318160-22318790	585	194	22.72	6.12	Cytoplasmic	Minus
*GrGSTU25*	Gorai.011G163600.1	Chr11: 31290617-31297277	675	224	26.10	5.92	Cytoplasmic	Plus
*GrGSTU26*	Gorai.012G099100.1	Chr12: 21014065-21015298	663	220	25.46	6.03	Cytoplasmic	Minus
*GrGSTU27*	Gorai.012G120900.1	Chr12: 27604435-27605617	705	234	26.09	6.45	Cytoplasmic	Minus
*GrGSTU28*	Gorai.012G121000.1	Chr12: 27610911-27612136	705	234	25.95	5.36	Chloroplast	Minus
*GrGSTU29*	Gorai.012G121100.1	Chr12: 27617631-27619108	705	234	25.92	5.25	Chloroplast	Minus
*GrGSTU30*	Gorai.012G121400.1	Chr12: 27665597-27666786	711	236	26.16	5.76	Cytoplasmic	Plus
*GrGSTU31*	Gorai.013G014600.1	Chr13: 997489-999065	663	220	24.48	5.26	Cytoplasmic	Plus
*GrGSTU32*	Gorai.013G112700.1	Chr13: 26930123-26930852	660	219	25.63	7.17	Cytoplasmic	Plus
*GrGSTU33*	Gorai.013G113600.1	Chr13: 27659031-27660061	681	226	25.74	5.29	Cytoplasmic	Plus
*GrGSTU34*	Gorai.013G177200.1	Chr13: 46961394-46962365	660	219	25.17	6.53	Chloroplast	Minus
*GrGSTU35*	Gorai.013G177300.1	Chr13: 47002219-47003549	660	219	25.46	6.84	Cytoplasmic	Plus
*GrGSTU36*	Gorai.013G177400.1	Chr13: 47013066-47013998	648	215	24.89	6.25	Cytoplasmic	Plus
*GrGSTU37*	Gorai.013G177600.1	Chr13: 47037500-47039519	660	219	25.43	5.58	Cytoplasmic	Plus
*GrGSTU38*	Gorai.013G179300.1	Chr13: 47275362-47277951	660	219	25.44	6.9	Cytoplasmic	Minus
*GrGSTF1*	Gorai.001G083600.1	Chr01: 8834222-8836505	645	214	24.58	5.76	Cytoplasmic	Plus
*GrGSTF2*	Gorai.004G141900.1	Chr04: 40004892-40006445	648	215	24.33	6.19	Cytoplasmic	Plus
*GrGSTF3*	Gorai.007G129400.1	Chr07: 10398983-10400208	660	219	24.79	5.42	Cytoplasmic	Minus
*GrGSTF4*	Gorai.007G175100.1	Chr07: 16220916-16222730	648	215	24.79	5.34	Cytoplasmic	Minus
*GrGSTF5*	Gorai.007G240200.1	Chr07: 33067227-33069528	660	219	24.68	8.35	Cytoplasmic	Plus
*GrGSTF6*	Gorai.008G010200.1	Chr08: 1197081-1198882	648	215	24.07	6.13	Cytoplasmic	Plus
*GrGSTF7*	Gorai.011G211600.1	Chr11: 51061907-51062856	697	198	22.30	5.82	Cytoplasmic	Plus
*GrGSTT1*	Gorai.004G211100.1	Chr04: 54407517-54410688	837	278	31.95	9.52	Mitochondrial	Plus
*GrGSTT2*	Gorai.007G023300.1	Chr07: 1661709-1664000	756	251	28.50	9.37	Cytoplasmic	Plus
*GrGSTT3*	Gorai.008G246300.1	Chr08: 53071831-53074716	753	250	28.31	9.49	Cytoplasmic	Plus
*GrGSTZ1*	Gorai.008G114200.1	Chr08: 34672415-34675860	774	257	28.90	5.72	Cytoplasmic	Minus
*GrGSTZ2*	Gorai.011G090100.1	Chr11: 9558249-9561007	657	218	24.73	5.21	Cytoplasmic	Minus
*GrGSTL1*	Gorai.002G252300.1	Chr02: 61557665-61560819	738	245	28.26	5.32	Cytoplasmic	Minus
*GrGSTL2*	Gorai.006G177600.1	Chr06: 43499453-43502034	714	237	26.93	5.12	Cytoplasmic	Plus
*GrGSTL3*	Gorai.013G024200.1	Chr13: 1770849 1774598	969	322	36.58	7.6	Chloroplast	Plus
*GrEF1Bγ1*	Gorai.008G046200.1	Chr08: 6226741-6230087	1266	421	47.69	7.53	Cytoplasmic	Minus
*GrEF1Bγ2*	Gorai.008G046300.1	Chr08: 6259415 6262692	1266	421	47.69	7.53	Cytoplasmic	Minus
*GrDHAR1*	Gorai.001G089300.1	Chr01: 9656330-9658909	789	262	29.24	8.79	Chloroplast	Minus
*GrDHAR2*	Gorai.011G246200.1	Chr11: 57230753-57234321	639	212	23.54	6.17	Cytoplasmic	Plus
*GrDHAR3*	Gorai.012G068600.1	Chr12: 10022598-10024825	639	212	23.47	6.96	Cytoplasmic	Minus
*GrTCHQD1*	Gorai.013G108000.1	Chr13: 23436664 23439362	798	265	30.94	9.27	Nuclear	Minus

**Table 2:** **The information of 49 *GST* genes from *G. arboreum***.

**Gene name**	**Gene identifier**	**Genomics position**	**CDS**	**Size(AA)**	***Mw(kDa)***	***pI***	**Preditced subcellular localization**	**Strand**
*GaGSTU1*	Cotton_A_37696	Chr01: 115962251-115962956	612	203	23.12	5.42	Cytoplasmic	Plus
*GaGSTU2*	Cotton_A_05897	Chr01: 141131040-141131830	687	228	26.56	5.79	Cytoplasmic	Minus
*GaGSTU3*	Cotton_A_36350	Chr04: 36480986-36481718	663	220	25.34	5.69	Cytoplasmic	Plus
*GaGSTU4*	Cotton_A_19149	Chr04: 77946780-77947175	396	131	15.40	9.48	Mitochondrial	Plus
*GaGSTU5*	Cotton_A_24534	Chr05: 56685154-56687068	675	224	26.24	5.92	Cytoplasmic	Minus
*GaGSTU6*	Cotton_A_24526	Chr05: 56775166-56776607	678	225	26.59	6.56	Cytoplasmic	Minus
*GaGSTU7*	Cotton_A_24525	Chr05: 56779832-56780826	675	224	26.33	6.08	Cytoplasmic	Minus
*GaGSTU8*	Cotton_A_24523	Chr05: 56804944-56807155	672	223	25.82	5.88	Cytoplasmic	Minus
*GaGSTU9*	Cotton_A_24522	Chr05: 56808079-56810315	672	223	25.77	5.1	Cytoplasmic	Minus
*GaGSTU10*	Cotton_A_27775	Chr07: 34539787-34541185	639	212	24.72	5.82	Cytoplasmic	Minus
*GaGSTU11*	Cotton_A_27774	Chr07: 34545395-34546394	711	236	27.46	7.84	Cytoplasmic	Minus
*GaGSTU12*	Cotton_A_34756	Chr09: 44598342-44600496	798	265	30.51	5.18	Cytoplasmic	Minus
*GaGSTU13*	Cotton_A_29359	Chr10: 83946177-83947060	699	232	26.86	8.68	Cytoplasmic	Minus
*GaGSTU14*	Cotton_A_29361	Chr10: 84019904-84020941	519	172	19.84	8.56	Cytoplasmic	Minus
*GaGSTU15*	Cotton_A_29364	Chr10: 84167770-84168839	663	220	25.70	5.42	Cytoplasmic	minus
*GaGSTU16*	Cotton_A_02462	Chr11: 21179427-21180580	651	216	24.69	6.14	Cytoplasmic	Minus
*GaGSTU17*	Cotton_A_02461	Chr11: 21188528-21189567	678	225	25.85	5.75	Cytoplasmic	Minus
*GaGSTU18*	Cotton_A_02460	Chr11: 21222441-21223570	771	256	29.17	7.6	Cytoplasmic	Minus
*GaGSTU19*	Cotton_A_35955	Chr11: 46995035-46995773	663	220	25.92	6.13	Cytoplasmic	Minus
*GaGSTU20*	Cotton_A_03413	Chr12: 26107909-26108689	711	236	26.15	5.77	Cytoplasmic	Minus
*GaGSTU21*	Cotton_A_03415	Chr12: 26129084-26129876	705	234	26.11	5.53	Chloroplast	Plus
*GaGSTU22*	Cotton_A_03416	Chr12: 26135225-26136027	705	234	25.81	5.73	Cytoplasmic	Plus
*GaGSTU23*	Cotton_A_21914	Chr13: 56682472-56683218	660	219	25.09	6.53	Cytoplasmic	Plus
*GaGSTU24*	Cotton_A_21915	Chr13: 56711009-56711617	522	173	20.17	7.62	Cytoplasmic	Plus
*GaGSTU25*	Cotton_A_21916	Chr13: 56723260-56724010	660	219	25.57	6.91	Cytoplasmic	Plus
*GaGSTU26*	Cotton_A_21917	Chr13: 56759324-56760726	660	219	25.41	5.58	Cytoplasmic	Plus
*GaGSTU27*	Cotton_A_21938	Chr13: 57062669-57063763	612	203	23.53	8.47	Cytoplasmic	Minus
*GaGSTU28*	Cotton_A_01020	Chr13: 74881033-74882362	669	222	24.74	5.27	Chloroplast	Minus
*GaGSTU29*	Cotton_A_35457	Chr13: 88582069-88582846	675	224	25.49	5.28	Cytoplasmic	Minus
*GaGSTF1*	Cotton_A_12201	Chr01: 84548715-84550731	645	214	24.57	5.97	Cytoplasmic	Minus
*GaGSTF2*	Cotton_A_22529	Chr03: 75186297-75187194	648	215	24.34	6.05	Cytoplasmic	Minus
*GaGSTF3*	Cotton_A_12321	Chr04: 23049823-23051175	648	215	24.77	5.46	Cytoplasmic	Minus
*GaGSTF4*	Cotton_A_23310	Chr04: 26934603-26935467	660	219	24.83	5.41	Cytoplasmic	Plus
*GaGSTF5*	Cotton_A_34119	Chr07: 36371353-36372180	660	219	26.64	8.67	Nuclear	Minus
*GaGSTF6*	Cotton_A_34451	Chr11: 48647504-48648924	648	215	24.09	6.44	Cytoplasmic	Minus
*GaGSTT1*	Cotton_A_25983	Chr03: 24938613-24940995	753	250	28.73	8.99	Cytoplasmic	Minus
*GaGSTT2*	Cotton_A_14469	Chr07: 47840336-47842724	753	250	28.39	9.45	Cytoplasmic	Plus
*GaGSTT3*	Cotton_A_02311	Chr11: 22362230-22367274	753	251	28.37	8.79	Cytoplasmic	Plus
*GaGSTZ1*	Cotton_A_40149	Chr08: 87761510-87763550	648	215	24.37	5.34	Cytoplasmic	Plus
*GaGSTZ2*	Cotton_A_03363	Chr12: 24422813-24429189	1245	414	47.39	5.6	Plasma Membrane	Minus
*GaGSTL1*	Cotton_A_00425	Chr02: 69229452-69232705	717	238	27.46	5.32	Cytoplasmic	Minus
*GaGSTL2*	Cotton_A_02453	Chr11: 21297228-21299341	774	257	29.24	4.82	Cytoplasmic	Minus
*GaGSTL3*	Cotton_A_00921	Chr13: 73890473-73893510	969	322	36.45	7	Chloroplast	Minus
*GaEF1Bγ1*	Cotton_A_17724	Chr06: 28533801-28535983	1263	420	47.65	7.53	Cytoplasmic	Plus
*GaEF1Bγ2*	Cotton_A_17725	Chr06: 28612381-28614486	1179	392	44.63	6.22	Cytoplasmic	Plus
*GaDHAR1*	Cotton_A_30812	Chr01: 130018051-130020359	789	262	29.32	7.67	Chloroplast	Minus
*GaDHAR2*	Cotton_A_15148	Chr07: 100493251-100495337	639	212	23.44	6.39	Cytoplasmic	Minus
*GaDHAR3*	Cotton_A_15919	Chr12: 9370070-9371802	639	212	23.50	7.69	Cytoplasmic	Plus
*GaTCHQD1*	Cotton_A_36105	Chr08: 121004224-121005417	831	276	32.16	9.05	Nuclear	Minus

In addition to full-length *GST* genes, 16 partial *GST* genes and 4 other *GST* genes belong to two other subfamilies (2 of mPGES2 subfamily and 2 of C_omega_like subfamily) that distinct from the canonical GST were identified in *G. raimondii* genome (Supplementary Table 2). The *G. arboreum* genome also contains 12 partial GST fragments and one mPGES2 and two C_omega_like genes respectively (Supplementary Table 1). Domain structure analyses revealed that these partial *GST* genes contained only GST N- or C- domain or both of partial domains. Due to their small size, we were unable to analyze them in the subsequent research.

To reveal the classes of *G. raimondii* GSTs and *G. arboreum* GSTs, all these full-length GST protein sequences were initially subjected to National Center for Biotechnology Information's (NCBI) Conserved Domain Database (Marchler et al., 2014). Results shown that all the putative GSTs of the two cotton species can be divided into eight subgroups as Tau, Phi, Theta, Zeta, Lambda, EF1Bγ, DHAR, and TCHQD. According to the proposed nomenclature for *GST* genes (Dixon et al., 2002; Dixon and Edwards, 2010), all these *GST* genes were designated as *GrGSTs* for *G. raimondii* and *GaGSTs* for *G. arboreum*. The genes of subgroups belong to Tau, Phi, Theta, Zeta, Lambda, EF1Bγ, DHAR, and TCHQD were named as *GSTU, GSTF, GSTT, GSTZ, GSTL, EF1B*γ, *DHAR*, and *TCHQD*, respectively, followed by a gene number. The numbering of each subgroup *GST* genes was based on their position from top to the bottom on each corresponding chromosome and different chromosomes from chromosome 1 to chromosome 13. Though the *G. arboreum* genome was almost two times larger than the *G. raimondii* (Paterson et al., 2012; Wang et al., 2012; Li et al., 2014), there were only 49 *GST* genes identified from *G. arboreum*, 10 genes less than that of the *G. raimondii*. The length, molecular weight (Mw), isoelectric points (pI), and the predicted subcellular localization of the 59 *GrGSTs* and 49 *GaGSTs* were deduced from their predicted protein sequences. For *G. raimondii*, the amino acid numbers encoded from the identified *GST* genes varied from 194 of GrGSTU24 to 421 of GrEF1Bγ1 and GrEF1Bγ2, and their molecular weight ranged between 22.25 kDa of GrGSTU17 to 47.69 kDa of GrEF1Bγ1 and GrEF1Bγ2. Similarly, the molecular weight of GaGST proteins ranged from 15.40 kDa of GaGSTU4 to 47.65 kDa of GaEF1Bγ1, and the amino acid numbers varied from 131 of GaGSTU4 to 420 of GaEF1Bγ1. Protein subcellular localization is important for understanding its function (Chou and Shen, 2007). Most GrGUSTs and GaGSTs were predicted to be located in the cytoplasm, only a small parts were predicted to be in the mitochondria, chloroplast, or nuclear.

### Phylogenetic analysis of the *GST* gene family

**Figure 1:**
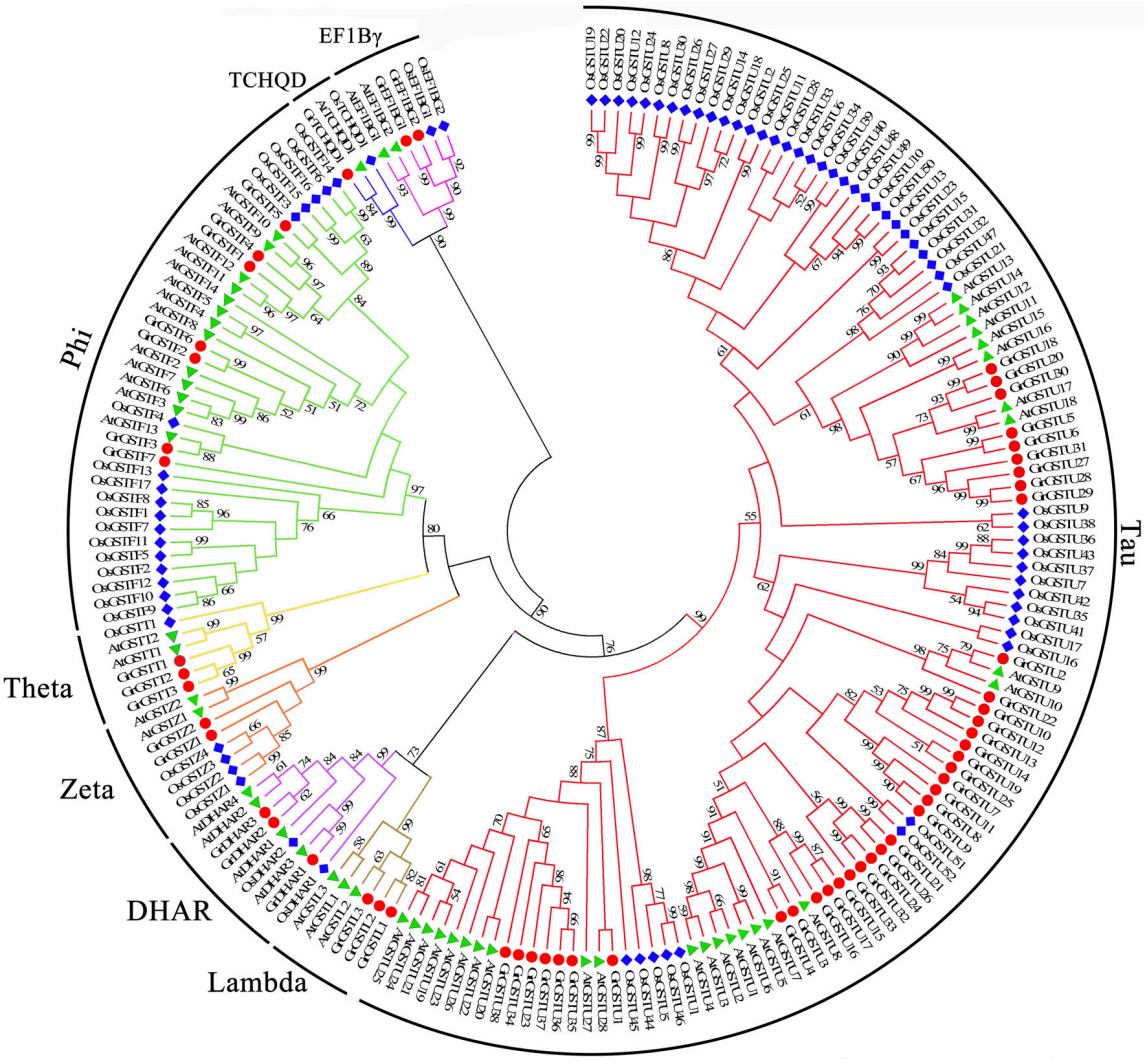
**Phylogenetic relationships of *GST* genes from *G. raimondii*, *Arabidopsis*, and rice**. The unrooted phylogentic tree was constructed using MEGA 5.2 by Neighbor-Joining method and the bootstrap test was performed with 1000 replicates. Percentage bootstrap scores of >50% were displayed. The *GST* genes from *G. raimondii, Arabidopsis* and rice were marked with the red dots, green triangles, and blue rhombuses respectively. And the branches of each subfamily were indicated in a specific color.

**Figure 2:**
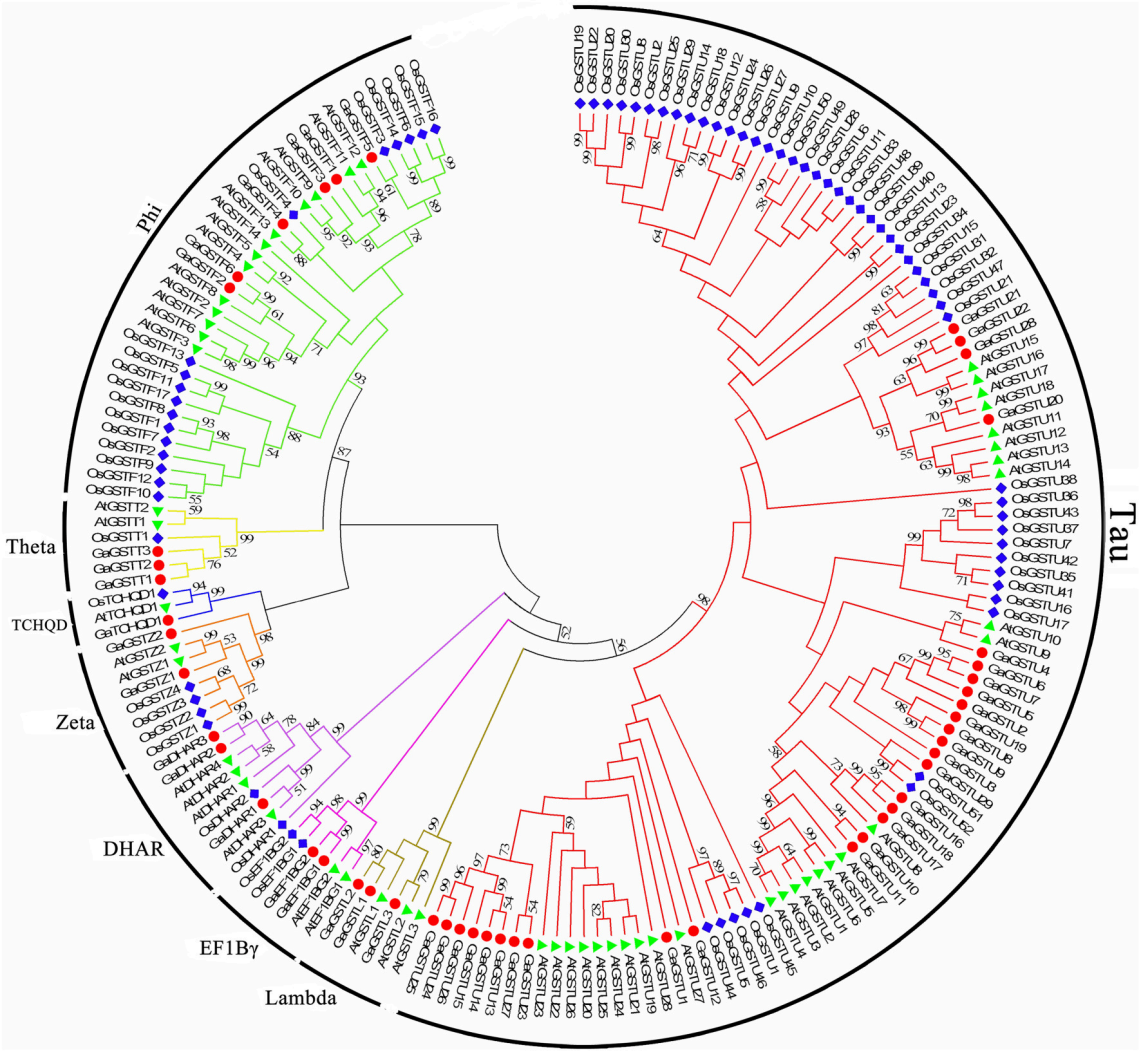
**Phylogenetic relationships of *GST* genes from *G. arboreum*, *Arabidopsis*, and rice**. The unrooted phylogentic tree was constructed using MEGA 5.2 by Neighbor-Joining method and the bootstrap test was performed with 1000 replicates. Percentage bootstrap scores of >50% were displayed. The *GST* genes from *G. arboreum, Arabidopsis* and rice were marked with the red dots, green triangles, and blue rhombuses, respectively. And the branches of each subfamily were indicated in a specific color.

To detect the phylogenetic relationship between *GST* genes, all the putative GSTs from two cotton species, as well as the GST proteins from Arabidopsis and rice, were aligned to generate an unrooted phylogenetic tree separately with Neiboring-Joining method (Figures 1, 2). Meanwhile, the phylogenetic trees reconstructed with Minimum Evolution method were almost identical with only minor differences at some branches (Supplementary Figures 1, 2), suggesting that the two methods were largely consistent with each other. The phylogenetic trees shown that GST proteins from *G. raimondii* or *G. arboreum, Arabidopsis*, and rice belonging to the same class were clustered together. It suggested that both the *GST* gene family of the two cotton species can be grouped into eight classes. The phylogenetic classification completely matched the classification based on NCBI CDD. It could be shown in Figure 1 that Tau contained the largest number of *GrGST* genes (38) followed by Phi (7). This phenomenon was correspond to *GST* genes in other plant species (Sappl et al., 2009; Jain et al., 2010; Chi et al., 2011). The plant specific Tau and Phi *GSTs* were inducible in plants when they were exposure to biotic and abiotic stresses (Nutricati et al., 2006). Other two plant specific GSTs classes, Lambda and DHAR were the only GSTs shown to be active as monomers (Mohsenzadeh et al., 2011). Theta class has a putative role in detoxifying oxidized lipids (Wagner et al., 2002). *G. raimondii* had three members in each of them. Among the four representative plant species, only rice lack the Lambda GSTs. There were two *GrGST* genes each in Zeta and EF1Bγ class, which functions in tyrosine catabolism and the encoding of γ subunit of eukaryotic translation elongation factor. As with the unusual class of *GST* gene family, TCHQD, only one member existed in *G. raimondii, Arabidopsis*, and rice. All the *GrGSTs* clustered with their *Arabidopsis* and rice counterparts. It suggested that the *GrGST* genes duplicated after the divergence of *G. raimondii, Arabidopsis*, and rice.

Similarly, 49 *GaGSTs* can be grouped into the eight classes (Figure 2), Tau had the largest number of GST genes (29), followed by Phi (6). There were three GST genes each in Lambda, DHAR, and Theta, two each in Zeta and EF1Bγ, and one in TCHQD.

### Orthologous relationships between *GrGSTs* and *GaGSTs*

**Figure 3:**
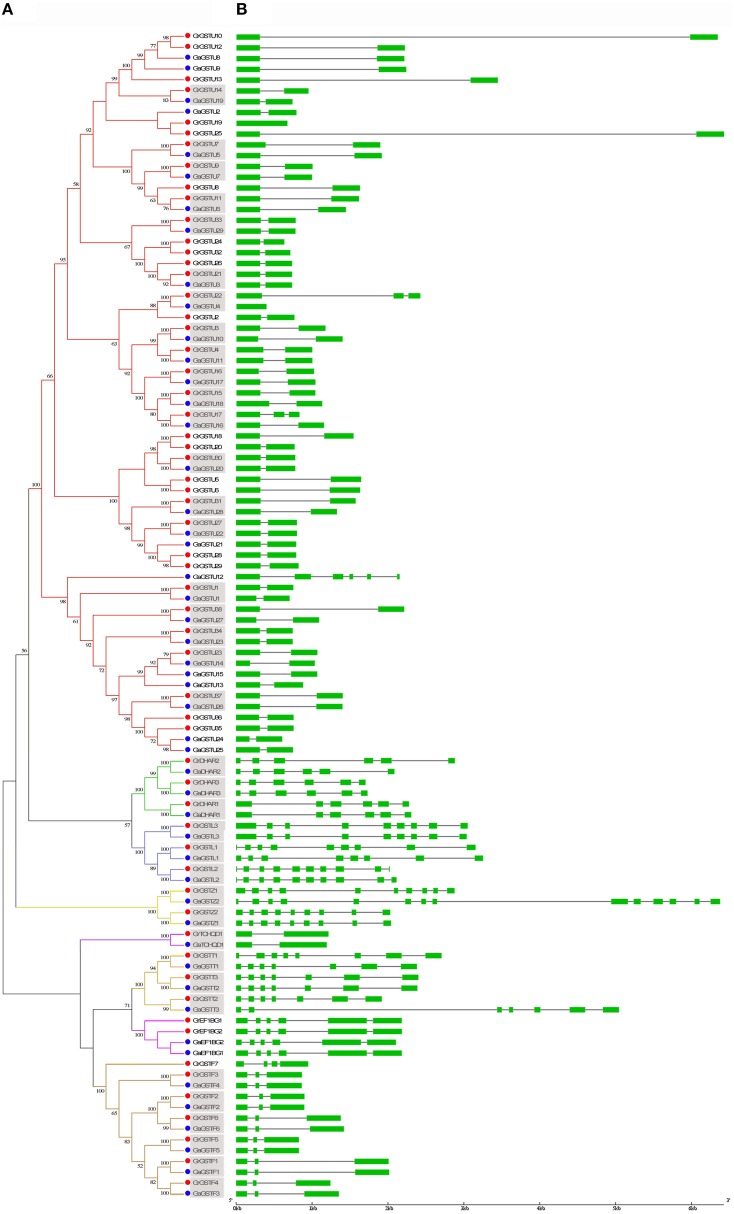
**Phylogenetic relationships and gene structure of *GST* genes from *G. raimondii* and *G. arboreum***. **(A)** The phylogenetic tree of all *GST* genes in *G. raimondii* and *G. arboreum* was constructed using MEGA 5.2 by Neighbor-Joining method and the bootstrap test was performed with 1000 replicates. Percentage bootstrap scores of >50% were displayed. The *GST* genes from *G. raimondii* and *G. arboreum* were marked with red dots and blue dots, respectively. Gene names in gray background shown orthologous pairs. **(B)** The exon-inton structure of *GST* genes from *G. raimondii* and *G. arboreum*. Exons were represented by green boxes and introns by gray lines.

In order to reveal the orthologous relationships among the members of *GST* gene family between *G. raimondii* and *G. arboreum*, the protein sequences of 59 predicted full-length *GrGST* genes and 49 predicted full-length *GaGST* genes were further applied to construct a separate unrooted phylogenetic tree (Figure 3A). The topology of the tree indicated that there were 38 pairs of orthologous genes between *G. raimondii* and *G. arboreum*, since these *GST* genes from the two cotton species respectively were in the terminal branches with high bootstrap values. However, the others were divergent apparently, the orthologous relationships among them could not be confirmed. Among the most abundant Tau class in *G. raimondii* and *G. arboreum*, only 20 orthologous gene pairs were found. Whereas, Phi class harbored 6 pairs of orthologous genes apart from *GrGSTF7*. All the *GST* genes in DHAR (3 pairs), Lambda (3 pairs), Zeta (2 pairs), Theta (3 pairs), and TCHQD (1 pair) were in the adjacent clades separately, suggesting that all of them in each class were orthologous genes. The orthologous relationships between the two *Gossypium* species were displayed in Supplementary Figure 3. However, all the EF1Bγ genes were of paralogous. Moreover, there were several pairs of paralogous genes in the Tau subfamily both in *G. raimondii* and *G. arboreum*, since the genes from the same genome were in the terminal branches of the phylogenetic tree.

### Gene structure of *GrGSTs* and *GaGSTs*

To investigate the possible structural evolution of *GST* gene family in the two diploid cotton species, the gene structures of *GrGSTs* and *GaGSTs* were compared separately. The details of the comparison were illustrated in Figure 3B. In general, the exon/intron organizations of *GSTs* were consistent with the phylogenetic subfamilies showed in Figure 3A. And the gene structures were conserved within the same group. As an example, a host of *GST* genes in Tau possessed one intron, except for *GaGSTU12*, which contained five introns. Most members of Phi class had two introns, except for *GrGSTF7* which had three introns. All the members in Theta class possessed six introns except *GrGST1* that had seven. The structures of the genes in DHAR, TCHQD, and EF1Bγ classes were relatively highly conserved, and every gene in the same group had the same intron number. In contrast, the exon/intron distribution patterns in the genes of Lambda class were various. The intron numbers ranged from seven of *GaGSTL1* to nine of *GaGSTL2* and *GrGSTL2*. Zeta class also displayed great variability in gene structures. *GaGSTZ2* had 13 introns, which is the maximum in all the *GST* genes, and *GrGSTZ1* contained nine introns, while *GrGSTZ2* and *GaGSTZ1* each had eight introns. As expected, the gene structures of orthologous pairs were almost identical with only minor differences with the exception of *GrGSTU22*/*GaGSTU4, GrGSTU17*/*GaGSTU16, GrGSTL1*/*GaGSTL1, GrGSTZ1*/*GaGSTZ1*, and *GrGSTT1*/*GaGSTT1*. Additionally, the gene structures among the orthologous pairs were uniformly observed in Phi and EF1Bγ classes.

### Chromosomal localization and gene duplication

**Figure 4:**
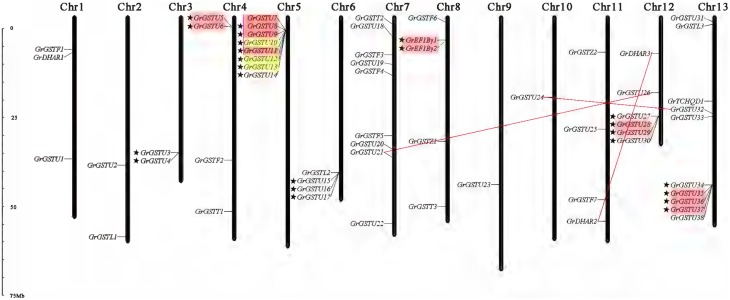
**Chromosomal distribution and gene duplication of *GST* genes in *G. raimondii***. The chromosome number was indicated at the top of each chromosome representation. The scale on the left was in megabases (Mb). The genes with a pentagram left represent *GST* gene clusters. The tandem duplicated genes were highlighted with outlined boxes. And the segmental duplicated gene pairs are connected with red lines.

The 59 non-redundant *GrGST* genes were mapped on the 13 *G. raimondii* chromosomes (Figure 4). Normally, the number of *GrGST* genes on each chromosome varied widely. Chromosome 13 contained 10 *GST* genes, followed by chromosome 7 and chromosome 5 on which nine and eight members were found, respectively. Chromosome 12 had six genes, and chromosome eight had five. Chromosome 4, Chromosome 6, and Chromosome 11 contained four genes each. There were three *GST* genes on Chromosome 1. Both chromosome 2 and chromosome 3 harbored two genes, whereas each only single *GST* gene was localized on chromosome 9 and chromosome 10. Obviously, they were distributed unevenly among 13 chromosomes. In addition, most of the *GrGST* genes in Tau class were clustered on chromosomes. Referred to the criterion of tandem duplication (Zhao et al., 2014; Qiao et al., 2015), we defined gene cluster as that two adjacent *GST* genes were separated by a maximum of five intervening genes. A total of seven gene clusters were detected on seven different chromosomes, and six of them were from tau class (22 genes) and the other was produced by the genes that from EF1Bγ class (two genes). It had been revealed that tandem duplication and/or segmental duplication played a significant role in the generation of gene families. To elucidate the expanded mechanism of *GST* gene family in *G. raimondii*, the gene duplication events were investigated, and 12 tandem duplication events, *GrGSTU5*/*GrGSTU6, GrGSTU7*/*GrGSTU11, GrGSTU8*/*GrGSTU7, GrGSTU9*/*GrGSTU8, GrGSTU10*/ *GrGSTU12, GrGSTU12*/*GrGSTU13, GrGSTU13*/*GrGSTU10, GrGSTU28*/*GrGSTU29, GrGSTU35*/*GrGSTU36, GrGSTU36*/*GrGSTU37, GrGSTU37*/*GrGSTU35*, and *GrEF1B*γ*1*/*GrEF1B*γ*2*, were detected in the *G. raimondii* genome (Figure 4). Interestingly, all tandem duplicated gene pairs were concluded in their different gene clusters respectively. Furthermore, three segmental duplication events, *GrGSTU21*/*GrGSTU26, GrGSTU24*/*GrGSTU32*, and *GrDHAR2*/*GrDHAR3*, were detected. It suggested that both the two kinds of duplication events contributed to the *GST* gene family expansion in *G. raimondii*.

**Figure 5:**
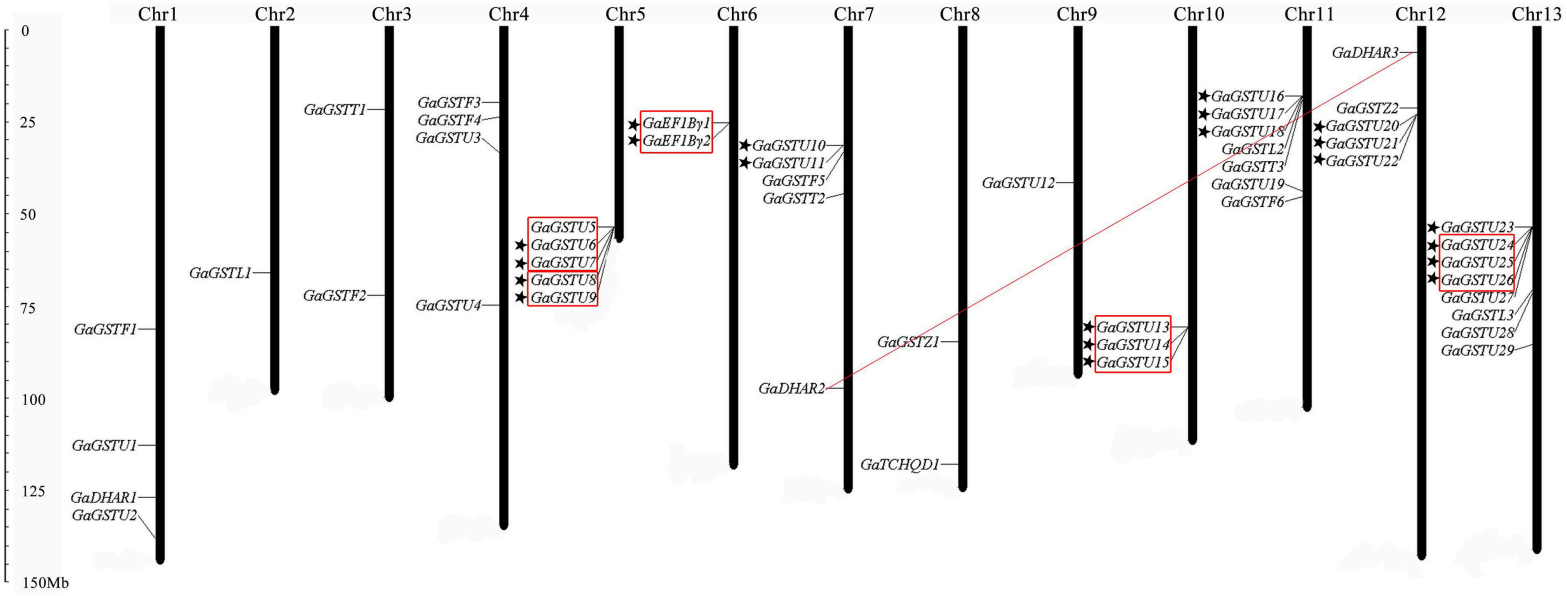
**Chromosomal distribution and gene duplication of *GST* genes in *G. arboreum***. The chromosome number was indicated at the top of each chromosome representation. The scale on the left was in megabases (Mb). The genes with a pentagram left represent *GST* gene clusters. Red outlined boxes represent tandem duplicated genes. And the segmental duplicated gene pairs were connected with red lines.

Like the case in *G. raimondii*, the 49 *GaGST* gene loci distributed unevenly across the 13 chromosomes in *G. arboreum*, ranging from 1 to 8 genes per chromosome (Figure 5). A maximum number of eight genes were located on chromosome 13 closely, followed by seven genes on chromosome 11. In contrast, only one gene was located on chromosome 2 and chromosome 9 each. There were also seven gene clusters distributed on seven different chromosomes. Analogously, six out of the seven gene clusters were composed of 19 *GaGSTs* in Tau class and the rest one was formed by *GaEF1B*γ class. A total of 10 tandem duplication events and one segmental duplication event were found. We also concluded that the expansion of *GST* gene family in *G. arboreum* was mainly attributed to tandem duplication events rather than segmental duplication event.

### Selective pressure analysis of the duplicated *GST* genes

To investigate the selective constrains on duplicated *GST* genes, the non-synonymous to synonymous substitution ratio (Ka/Ks) for each pair of duplicated *GST* genes were calculated. Generally, Ka/Ks ratio >1 indicates positive selection, Ka/Ks = 1 indicates neutral selection, while a ratio < 1 indicates negative or purifying selection. In this study, 15 duplicated pairs in the *G. raimondii* and 11 duplicated pairs in *G. arboreum GST* gene family were investigated respectively. In *G. raimondii*, the Ka/Ks ratio for 11 duplicated pairs were < 1 (Table 3), with most of them being even < 0.3, which suggested that they had experienced strong purifying selection pressure. However, the remaining four duplicated pairs with ratios >1 seemed to be under positive selection. While in the case of *G. arboreum*, 10 out of 11 duplicated pairs had undergone purifying selection pressure, and only one pair of duplicated *GST* genes with a ratio >1 were found in *G. arboreum*. Those observations reflected that the functions of the duplicated *GST* genes in the two cottons did not diverge much during subsequent evolution. And the purifying selection might contribute largely to the maintenance of function in *G. arboreum* GST family.

**Table 3:** **Ka/Ks analysis for the duplicated *GST* gene pairs from *G. raimondii* and *G. arboretum***.

**Species**	**Duplicated gene 1**	**Duplicated gene 2**	**Ka**	**Ks**	**Ka/Ks**	**Purifying selection**	**Duplicate type**
*G. raimondii*	*GrGSTU5*	*GrGSTU6*	0.016	0.036	0.451	Yes	Tandem
	*GrGSTU7*	*GrGSTU11*	0.126	0.463	0.271	Yes	Tandem
	*GrGSTU8*	*GrGSTU7*	0.102	0.380	0.269	Yes	Tandem
	*GrGSTU9*	*GrGSTU8*	0.043	0.305	0.142	Yes	Tandem
	*GrGSTU10*	*GrGSTU12*	0.042	0.083	0.513	Yes	Tandem
	*GrGSTU12*	*GrGSTU13*	0.100	0.293	0.340	Yes	Tandem
	*GrGSTU13*	*GrGSTU10*	0.083	0.023	3.544	No	Tandem
	*GrGSTU21*	*GrGSTU26*	0.016	0.007	2.275	No	Segmental
	*GrGSTU24*	*GrGSTU32*	0.054	0.044	1.227	No	Segmental
	*GrGSTU28*	*GrGSTU29*	0.009	0.019	0.489	Yes	Tandem
	*GrGSTU35*	*GrGSTU36*	0.020	0.015	1.361	No	Tandem
	*GrGSTU36*	*GrGSTU37*	0.085	0.290	0.291	Yes	Tandem
	*GrGSTU37*	*GrGSTU35*	0.080	0.292	0.276	Yes	Tandem
	*GrDHAR2*	*GrDHAR3*	0.084	0.501	0.168	Yes	Segmental
	*GrEF1Bγ1*	*GrEF1Bγ2*	0.004	0.022	0.187	Yes	Tandem
*G. arboreum*	*GaGSTU5*	*GaGSTU6*	0.121	0.463	0.261	Yes	Tandem
	*GaGSTU6*	*GaGSTU7*	0.064	0.421	0.153	Yes	Tandem
	*GaGSTU8*	*GaGSTU9*	0.059	0.097	0.615	Yes	Tandem
	*GaGSTU13*	*GaGSTU14*	0.071	0.164	0.431	Yes	Tandem
	*GaGSTU14*	*GaGSTU15*	0.065	0.187	0.350	Yes	Tandem
	*GaGSTU15*	*GaGSTU13*	0.048	0.170	0.280	Yes	Tandem
	*GaGSTU24*	*GaGSTU25*	0.015	0.009	1.591	No	Tandem
	*GaGSTU25*	*GaGSTU26*	0.082	0.328	0.251	Yes	Tandem
	*GaGSTU26*	*GaGSTU24*	0.098	0.308	0.316	Yes	Tandem
	*GaDHAR2*	*GaDHAR3*	0.077	0.462	0.166	Yes	Segmental
	*GaEF1Bγ1*	*GaEF1Bγ2*	0.008	0.040	0.194	Yes	Tandem

### The expression profiles of potential salt stress-responsive *GST* genes

Salt stress is one of the serious environmental stresses that most land plants might encounter during the process of their growth. Many GSTs have been implicated in various abiotic stress responses in plants (Droog, 1997; Scarponi et al., 2006; Sharma et al., 2014; Yang et al., 2014). However, little is known about the functions of *GST* genes in the cotton response to salt stress. The *cis*-elements in gene promoter regions might provide some indirect evidence for the functional dissection of *GST* genes in stress response (Zhou et al., 2013). Though the specific items of salt-responsive element were not existed in the PLACE database, some *cis*-elements might respond to multiple environment stimuli (Higo et al., 1999). All of putative environment stimulus responsive *cis*-elements in cotton *GST* genes were detected (Supplementary Table 3). The results revealed that the majority of *GST* genes, 19 *GrGSTs*, and 21 *GaGSTs*, contained relevant *cis*-elements in promoter sequences, which indicated that these cotton GST genes might the signal transduction of the plant response to salt stress.

**Figure 6:**
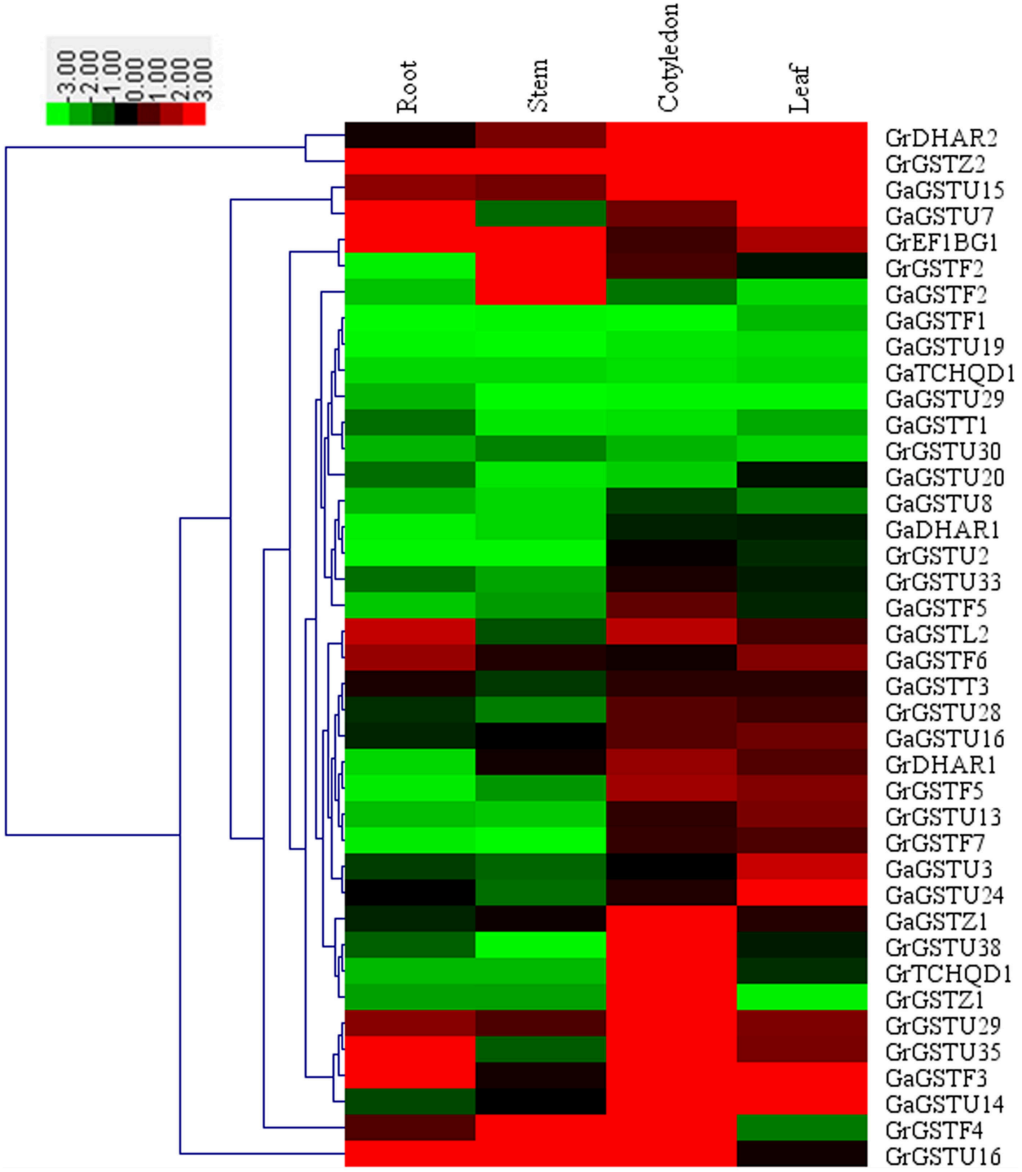
**Expression patterns of 40 selected *GST* genes in four representative tissues of *G. raimondii* and *G. arboreum***. The color bar represents the relative signal intensity values.

To verify the expression patterns of these *GST* genes, a comprehensive qRT-PCR analysis of 40 selected *GSTs* were performed (Figure 6). As shown in Figure 6, cotyledons and leaves exhibited more concentrated expression levels compared with roots and stems. Most of these 40 cotton *GST* genes had specific spatial expression patterns. *GrDHAR2, GaGSTU15, GaGSTF3*, and *GaGSTU14* preferentially expressed both in cotyledons and leaves, and *GrGSTZ2* were highly expressed in all tissues detected. There were seven orthologs among the selected genes, but only one pair *GrGSTU30*/*GaGSTU20* clustered together. It was inferred that the expression of *GST* orthologs between *G. raimondii* and *G. arboreum* have experienced divergence.

**Figure 7:**
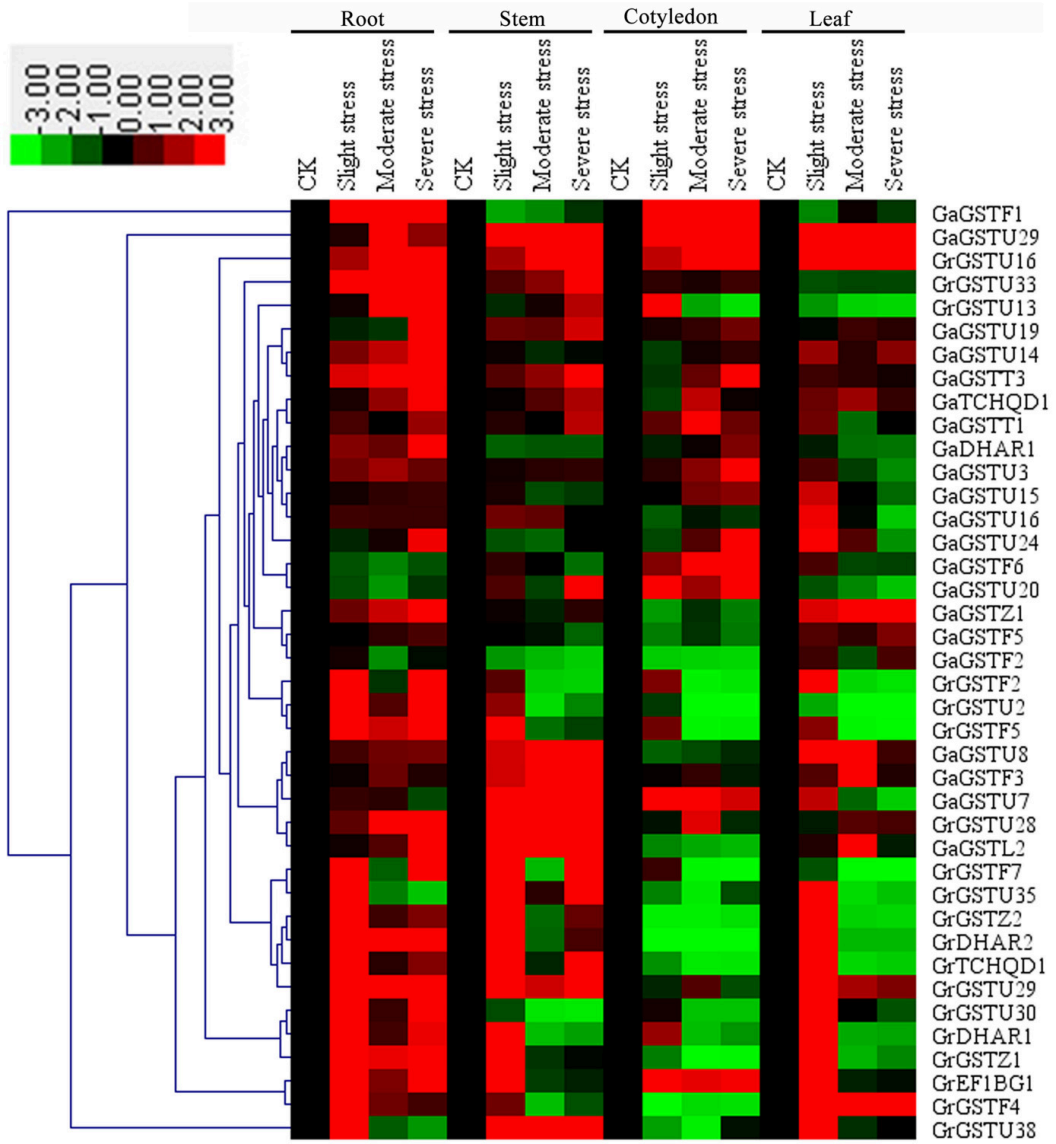
**Expression patterns of 40 selected *GST* genes in four representative tissues of *G. raimondii* and *G. arboreum* under salt stress**. The color bar represents the relative signal intensity values. The slight stress, moderate stress, and severe stress represent 50, 100, and 200 mM NaCl in *G. raimondii* and 100, 200, and 300 mM NaCl in *G. arboreum*, respectively.

The expression of all the selected *GST* genes has also been conducted in the roots, stems, cotyledons and leaves of two cotton species under salt treatments. Results showed altered expression patterns of either induction or suppression associated with at least one salt concentration (Figure 7). In roots, nearly all the selected *GST* genes showed up-regulated expression after salt treatment except for *GaGSTF6* and *GaGSTU20*. In stems, *GaGSTF1, GaDHAR1, GaGSTU24, GaGSTF2*, and *GrGSTU30* were down-regulated. Several *GST* genes displayed initial up-regulation and subsequent down-regulation. However, only a few up-regulated expressed *GST* genes were found in cotyledons compared with roots. *GaGSTF1, GaGSTU29, GaGSTU7*, and *GrEF1B*γ*1* showed insignificantly up-regulated expression by salt inducing in cotyledons. In leaves, the expressions of most selected *GST* genes were up-regulated just under slight salt stress. Nevertheless, *GaGSTU29, GrGSTU16, GaGSTZ1*, and *GrGSTF4* showed continued up-regulation in different level of salt stress. In addition, only one orthologs, *GaGSTF2*/*GrGSTF2*, were clustered together with similar expression patterns.

## Discussion

Salinity resulting mainly from NaCl is one of common environmental stresses that afflict the growth and yield of crops in many places of the world (Shabala, 2013). Salt stress may increase the reactive oxygen species (ROS) and damage the integrity of cell membranes, which triggering the disturbance of metabolism (Zhu, 2002). Plant adaptation to salt stress involved a series of biochemical pathways and lots of active compounds such as antioxidant enzymes (Guo et al., 2001).

### Phylogenetic analyses and evolution of *GST* gene family in *G. raimondii* and *G. arboreum*

A growing numbers of research works were devoted to elucidating the roles of plant GSTs in growth and stress responses (Oakley, 2005; Dixon et al., 2010; Skopelitou et al., 2012). A total of 59 and 49 putative *GST* genes were identified in the genomes of *G. raimondii* and *G. arboreum* respectively in present work. Phylogenetic analyses revealed that both *GrGSTs* and *GaGSTs* were more closely allied to *AtGSTs* than to *OsGSTs*, which were consistent with the evolutionary relationships among *G. raimondii, G. arboreum, Arabidopsis*, and rice. Moreover, all the *GST* genes from the three representative dicot species sorted into eight distinct clades, except the Lambda class which was absent in rice. This implied that these seven subfamilies but Lambda arose before divergence the monocots-dicots. In turns, Lambda group was either acquired after the evolutionary split of monocots-dicots or lost in rice. Intriguingly, the member of *GST* gene family in *G. raimondii* was a little bit more than that in *Arabidopsis* (55) and much less than that in rice (77). It was more obvious in the case of *G. arboreum* which contained the minimum *GST* genes among the four representative species, albeit the genomes of the two diploid cottons were larger than that of *Arabidopsis* and rice. A proper explanation of the phenomenon was that the transposable elements represented a major component of *Gossypium* genome (Hawkins et al., 2006).

### Tandem duplication plays a major role in the expansion of *GST* gene family in *G. raimondii* and *G. arboreum*

Like the *GST* gene families in other known plants (Soranzo et al., 2004; Sappl et al., 2009), most *GST* gene loci in *G. raimondii* and *G. arboreum* were present in genomes in clusters of two to seven genes. There were seven gene clusters each in the genomes of *G. raimondii* and *G. arboreum*, presumably as a result of multiple tandem duplication events in the common ancestor of *Gossypium*. The classifications of full-length *GST* genes suggested that whether in *G. raimondii* or *G. arboreum*, the members of Tau *GSTs* and Phi *GSTs* were more than the others. In addition, Tau *GSTs* occupied 12 of the total 14 *GST* gene clusters in the two diploid cottons. The rest of them were composed of EF1Bγ *GSTs*. It has been demonstrated that the expansion of gene families is mainly caused by gene duplication events, including tandem duplication, segmental duplication, transposition events, and whole-genome duplication (Blanc and Wolfe, 2004; Flagel and Wendel, 2009). It also could be speculated that the amplification of the *GST* gene family were mainly caused by the tandem duplication for Tau and Phi classes both in *G. raimondii* and *G. arboreum*. An intriguing finding was that purifying selection has predominated across the duplicated genes. The reasons might be that (1) deleterious mutations might occur in different domains in copies of genes with multiple independent domain subfunctions (Force et al., 1999; Lan et al., 2009); (2) purifying selection could eliminate deleterious loss-of-function mutations, thus fixed a new duplicate gene and enhanced the preservation of functional alleles at both duplicate loci (Tanaka et al., 2009). In addition, the number of *GaGST* genes was less than that of *GrGSTs*, although the genome size of *G. arboreum* is almost twice larger than that of *G. raimondii* (Paterson et al., 2012; Wang et al., 2012; Li et al., 2014). A theoretical explanation was that *G. arboreum* had undergone large-scale retrotransposons insertion during evolution (Li et al., 2014).

### Functional divergence of specific cotton *GST* genes under salt stress

It is worth to notice that orthologous *GST* gene pairs demonstrated very similar exon/intron distribution patterns in terms of exon length and intron number. However, the expression patterns of them were divergent. It might involve with the adaptation to different habitat of *G. raimondii* and *G. arboreum* after their species-specific evolution. Remarkably, the exons of the *GST* genes in the same classes were highly conserved whether of intraspecies or interspecies, but the introns were various with indel mutations (Xu et al., 2012). Further analyses are needed to elucidate the impacts of intron variation on gene function. The gene expression patterns can provide important clues for gene function. The tissue-specific expression patterns of 40 selected *GST* genes under normal condition reflected that they might play versatile functions in the growth and development of cotton. Additionally, they also shown divergent expression patterns under salt treatment. It is clear that the roots are the first tissues which salt stress directly affected in the soil or culture solution (Guo et al., 2001). In our study, what consistent with the fact proposed was that almost all the selected *GST* genes were up-regulated in response to salt stress in roots. By contrast, a majority of genes showed up-regulation in leaves only under slight salt stress. This was probably associated with the facts that these two tissues by themselves were distinct in structure and functions (Qing et al., 2009; Campo et al., 2014). Duplicate genes might have three different evolutionary fates, i.e., nonfunctionalization, subfunctionalization, and neofunctionalization (Liu et al., 2014). The expression pattern shifts of the duplicated genes *GaGSTU14*/*GaGSTU15* indicated the functional divergence after duplicated events. Among the seven orthologs, only one pair, *GrGSTF2*/*GaGSTF2*, clustered together under salt treatment. These findings further supported the assertion that expression divergence is often the first step in the functional divergence between duplicate genes, thereby increases the chance of duplicate genes being retained in a genome (Zhang, 2003).

In short, the *GST* gene family both in *G. raimondii* and *G. arboreum* were identified and characterized using bioinformatics approaches, and the results have provided a basis for further assessment of physiological roles of different *GST* genes in response to salt stress in *Gossypium* species.

## Author contributions

YD, SZ, and JC conceived all the experiments and analyzed data. YD performed experiments, drafted the manuscript and prepared the figures. YD and SZ wrote and reviewed the manuscript. CL and YZ prepared figures. QH analyzed data. JC performed the experiments. MD contributed to the manuscript preparation. All authors reviewed the manuscript.

### Conflict of interest statement

The authors declare that the research was conducted in the absence of any commercial or financial relationships that could be construed as a potential conflict of interest. The reviewer SR and Handling Editor declared their shared affiliation, and the Handling Editor states that the process nevertheless met the standards of a fair and objective review.

## Supplementary material

Supplementary Figure 1 **Phylogenetic analysis of *GST* genes from *G. raimondii, Arabidopsis*, and rice**. The unrooted phylogentic tree was constructed using MEGA 5.2 by Minimum Evolution method and the bootstrap test was performed with 1000 replicates. Percentage bootstrap scores of >50% were displayed. The *GST* genes from *G. raimondii, Arabidopsis* and rice were marked with the dots, triangles, and rhombuses, respectively.

Supplementary Figure 2 **Phylogenetic analysis of *GST* genes from *G. arboreum, Arabidopsis*, and rice**. The unrooted phylogentic tree was constructed using MEGA 5.2 by Minimum Evolution method and the bootstrap test was performed with 1000 replicates. Percentage bootstrap scores of >50% were displayed. The *GST* genes from *G. arboreum, Arabidopsis* and rice were marked with the dots, triangles, and rhombuses, respectively.

Supplementary Figure 3 **Locations and orthologs of *GST* genes in *G. raimondii* and *G. arboreum***. The picture was generated by Circos software. The chromosomes of *G. raimondii* and *G. arboreum* were shown with different colors and labeled as Gr and Ga, respectively. The putative orthologous genes belonging to the Tau, Phi, Lambda, DHAR, Zeta, Theta, and TCHQD1 subfamilies were connected by yellow, green, blue, purple, orange, red, and gray lines, respectively.

Supplementary Table 1 **PCR primers used in this study**.

Supplementary Table 2 **The information of partial *GST* genes and mPGES2 and C_pmega_lke subfamilies in *G. raimondii* and *G. arboreum***.

Supplementary Table 3 **The *cis*-elements involved in salt stress response in the promoter regions of *GrGSTs* and *GaGSTs***.

## References

[B1] Armstrong R. N. (1997). Structure, catalytic mechanism, and evolution of the glutathione transferases. Chem. Res. Toxicol. 10, 2–18. 10.1021/tx960072x 9074797

[B2] Bjellqvist B. Basse B. Olsen E. Celis J. E. (1994). Reference points for comparisons of two-dimensional maps of proteins from different human cell types defined in a pH scale where isoelectric points correlate with polypeptide compositions. Electrophoresis 15, 529–539. 10.1002/elps.1150150171 8055880

[B3] Blanc G. Wolfe K. H. (2004). Widespread paleopolyploidy in model plant species inferred from age distributions of duplicate genes. Plant Cell 16, 1667–1678. 10.1105/tpc.021345 15208399 PMC514152

[B4] Campo S. Baldrich P. Messeguer J. Lalanne E. Coca M. Segundo B. S. (2014). Overexpression of a calcium-dependent protein kinase confers salt and drought tolerance in rice by preventing membrane lipid peroxidation. Plant Physiol. 165, 688–704. 10.1104/pp.113.230268 24784760 PMC4044838

[B5] Chan C. Lam H. M. (2014). A putative lambda class glutathione S-transferase enhances plant survival under salinity stress. Plant Cell Physiol. 55, 570–579. 10.1093/pcp/pct201 24399237

[B6] Chi Y. Cheng Y. Vanitha J. Kumar N. Ramamoorthy R. Ramachandran S.. (2011). Expansion mechanisms and functional divergence of the glutathione s-transferase family in sorghum and other higher plants. DNA Res. 18, 1–16. 10.1093/dnares/dsq031 21169340 PMC3041506

[B7] Cho H.-Y. Yoo S.-Y. Kong K.-H. (2006). Cloning of a rice tau class GST isozyme and characterization of its substrate specificity. Pestic. Biochem. Physiol. 86, 110–115. 10.1016/j.pestbp.2006.02.003

[B8] Chou K. C. Shen H. B. (2007). Recent progress in protein subcellular location prediction. Anal. Biochem. 370, 1–16. 10.1016/j.ab.2007.07.006 17698024

[B9] Dixon D. P. Cummins I. Cole D. J. Edwards R. (1998). Glutathione-mediated detoxification systems in plants. Curr. Opin. Plant Biol. 1, 258–266. 10.1016/S1369-5266(98)80114-3 10066594

[B10] Dixon D. P. Edwards R. (2010). Glutathione transferases. Am. Soc. Plant Biol. 2010, e0131. 10.1199/tab.0131 22303257 PMC3244946

[B11] Dixon D. P. Lapthorn A. Edwards R. (2002). Plant glutathione transferases. Genome Biol. 3, 3004.1–3004.10. 10.1186/gb-2002-3-3-reviews3004 11897031 PMC139027

[B12] Dixon D. P. Skipsey M. Edwards R. (2010). Roles for glutathione transferases in plant secondary metabolism. Phytochemistry 71, 338–350. 10.1016/j.phytochem.2009.12.012 20079507

[B13] Droog F. (1997). Plant glutathione S-transferases, a tale of Theta and Tau. J. Plant Growth Regul. 16, 95–107. 10.1007/PL00006984

[B14] Edgar R. C. (2004). MUSCLE: a multiple sequence alignment method with reduced time and space complexity. BMC Bioinformatics 5:113. 10.1186/1471-2105-5-113 15318951 PMC517706

[B15] Edwards R. Dixon D. P. Walbot V. (2000). Plant glutathione S-transferases: enzymes with multiple functions in sickness and in health. Trends Plant. Sci. 5, 1360–1385. 10.1016/S1360-1385(00)01601-0 10785664

[B16] Finn R. D. Bateman A. Clements J. Coggill P. Eberhardt R. Y. Eddy S. R.. (2014). Pfam: the protein families database. Nucleic Acids Res. 42, D222–D230. 10.1093/nar/gkt1223 24288371 PMC3965110

[B17] Finn R. D. Tate J. Mistry J. Coggill P. C. Sammut S. J. Hotz H. R.. (2008). The Pfam protein families database. Nucleic Acids Res. 36, D281–D288. 10.1093/nar/gkr1065 18039703 PMC2238907

[B18] Flagel L. E. Wendel J. F. (2009). Gene duplication and evolutionary novelty in plants. New Phytol. 183, 557–564. 10.1111/j.1469-8137.2009.02923.x 19555435

[B19] Force A. Lynch M. Pickett F. B. Amores A. Yan Y. Postlethwait J. (1999). Preservation of duplicate genes by complementary, degenerative mutations. Genetics 151, 1531–1545. 10101175 10.1093/genetics/151.4.1531 PMC1460548

[B20] Frova C. (2006). Glutathione transferases in the genomics era: new insights and perspectives. Biomol. Eng. 23, 149–169. 10.1016/j.bioeng.2006.05.020 16839810

[B21] Galle A. Csiszar J. Secenji M. Guoth A. Cseuz L. Tari I.. (2009). Glutathione transferase activity and expression patterns during grain filling in flag leaves of wheat genotypes differing in drought tolerance: response to water deficit. J. Plant Physiol. 166, 1878–1891. 10.1016/j.jplph.2009.05.016 19615785

[B22] Grover C. E. Kim H. Wing R. A. Paterson A. H. Wendel J. F. (2007). Microcolinearity and genome evolution in the AdhA region of diploid and polyploid cotton (Gossypium). Plant J. 50, 995–1006. 10.1111/j.1365-313X.2007.03102.x 17461788

[B23] Guo Y. Halfter U. Ishitani M. Zhu J. K. (2001). Molecular characterization of functional domains in the protein kinase SOS2 that is required for plant salt tolerance. Plant Cell 13, 1383–1399. 10.1105/tpc.13.6.1383 11402167 PMC135579

[B24] Hawkins J. S. Kim H. Nason J. D. Wing R. A. Wendel J. F. (2006). Differential lineage-specific amplification of transposable elements is responsible for genome size variation in Gossypium. Genome Res. 16, 1252–1261. 10.1101/gr.5282906 16954538 PMC1581434

[B25] Higo K. Ugawa Y. Iwamoto M. Korenaga T. (1999). Plant cis-acting regulatory DNA elements (PLACE) database: 1999. Nucleic Acids Res. 27, 297–300. 10.1093/nar/27.1.297 9847208 PMC148163

[B26] Hu B. Jin J. Guo A. Y. Zhang H. Luo J. Gao G. (2015). GSDS 2.0: an upgraded gene feature visualization server. Bioinformatics 31, 1296–1297. 10.1093/bioinformatics/btu817 25504850 PMC4393523

[B27] Jain M. Ghanashyam C. Bhattacharjee A. (2010). Comprehensive expression analysis suggests overlapping and specific roles of rice glutathione S-transferase genes during development and stress responses. BMC Genomics 11:73. 10.1186/1471-2164-11-73 20109239 PMC2825235

[B28] Ji W. Zhu Y. Li Y. Yang L. Zhao X. Cai H.. (2010). Over-expression of a glutathione S-transferase gene, GsGST, from wild soybean (Glycine soja) enhances drought and salt tolerance in transgenic tobacco. Biotechnol. Lett. 32, 1173–1179. 10.1007/s10529-010-0269-x 20383560

[B29] Kadir Z. B. (1976). DNA evolution in the genus gossypium. Chromosoma (Berl.) 56, 85–94. 10.1007/BF00293732

[B30] Lan T. Yang Z. L. Yang X. Liu Y. J. Wang X. R. Zeng Q. Y. (2009). Extensive functional diversification of the Populus glutathione S-transferase supergene family. Plant Cell 21, 3749–3766. 10.1105/tpc.109.070219 19996377 PMC2814494

[B31] Larkin M. A. Blackshields G. Brown N. P. Chenna R. McGettigan P. A. McWilliam H.. (2007). Clustal W and Clustal X version 2.0. Bioinformatics 23, 2947–2948. 10.1093/bioinformatics/btm404 17846036

[B32] Letunic I. Doerks T. Bork P. (2015). SMART: recent updates, new developments and status in 2015. Nucleic Acids Res. 43, D257–D260. 10.1093/nar/gku949 25300481 PMC4384020

[B33] Li F. Fan G. Wang K. Sun F. Yuan Y. Song G.. (2014). Genome sequence of the cultivated cotton Gossypium arboreum. Nat. Genet. 46, 567–572. 10.1038/ng.2987 24836287

[B34] Librado P. Rozas J. (2009). DnaSP v5: a software for comprehensive analysis of DNA polymorphism data. Bioinformatics 25, 1451–1452. 10.1093/bioinformatics/btp187 19346325

[B35] Licciardello C. D'Agostino N. Traini A. Recupero G. R. Frusciante L. Chiusano M. L. (2014). Characterization of the glutathione S-transferase gene family through ESTs and expression analyses within common and pigmented cultivars of Citrus sinensis (L.) Osbeck. BMC Plant Biol. 14:39. 10.1186/1471-2229-14-39 24490620 PMC3922800

[B36] Liu W. Li W. He Q. Daud M. K. Chen J. Zhu S. (2014). Genome-wide survey and expression analysis of calcium-dependent protein kinase in Gossypium raimondii. PLoS ONE 9:e98189. 10.1371/journal.pone.0098189 24887436 PMC4041719

[B37] Liu Y. J. Han X. M. Ren L. L. Yang H. L. Zeng Q. Y. (2013). Functional divergence of the glutathione S-transferase supergene family in Physcomitrella patens reveals complex patterns of large gene family evolution in land plants. Plant Physiol. 161, 773–786. 10.1104/pp.112.205815 23188805 PMC3561018

[B38] Livak K. J. Schmittgen T. D. (2001). Analysis of relative gene expression data using real-time quantitative PCR and the 2(-Delta Delta C(T)) Method. Methods 25, 402–408. 10.1006/meth.2001.1262 11846609

[B39] Ma X. X. Jiang Y. L. He Y. X. Bao R. Chen Y. Zhou C. Z. (2009). Structures of yeast glutathione-S-transferase Gtt2 reveal a new catalytic type of GST family. EMBO Rep. 10, 1320–1326. 10.1038/embor.2009.216 19851333 PMC2799204

[B40] Maher C. Stein L. Ware D. (2006). Evolution of Arabidopsis microRNA families through duplication events. Genome Res. 16, 510–519. 10.1101/gr.4680506 16520461 PMC1457037

[B41] Marchler B. A. Derbyshire M. K. Gonzales N. R. Lu S. Chitsaz F. Geer L. Y.. (2014). CDD: NCBI's conserved domain database. Nucleic Acids. Res. 43, D222–D226. 10.1093/nar/gku1221 25414356 PMC4383992

[B42] Marrs K. A. (1996). The functions and regulation of glutathione S-transferases in plants. Annu. Rev. Plant Physiol. Plant Mol. Biol. 47, 127–158. 10.1146/annurev.arplant.47.1.127 15012285

[B43] Mohsenzadeh S. Esmaeili M. Moosavi F. Shahrtash M. Saffari B. Mohabatkar H. (2011). Plant glutathione S-transferase classification, structure and evolution. Afr. J. Biotechnol. 10, 8160–8165. 10.5897/AJB11.1024

[B44] Nutricati E. Miceli A. Blando F. De Bellis L. (2006). Characterization of two Arabidopsis thaliana glutathione S-transferases. Plant Cell Rep. 25, 997–1005. 10.1007/s00299-006-0146-1 16538523

[B45] Oakley A. J. (2005). Glutathione transferases: new functions. Curr. Opin. Struct. Biol. 15, 716–723. 10.1016/j.sbi.2005.10.005 16263269

[B46] Ouyang Y. Chen J. Xie W. Wang L. Zhang Q. (2009). Comprehensive sequence and expression profile analysis of Hsp20 gene family in rice. Plant Mol. Biol. 70, 341–357. 10.1007/s11103-009-9477-y 19277876

[B47] Paterson A. H. Wendel J. F. Gundlach H. Guo H. Jenkins J. Jin D.. (2012). Repeated polyploidization of Gossypium genomes and the evolution of spinnable cotton fibres. Nature 492, 423–427. 10.1038/nature11798 23257886

[B48] Qiao L. Zhang X. Han X. Zhang L. Li X. Zhan H.. (2015). A genome-wide analysis of the auxin/indole-3-acetic acid gene family in hexaploid bread wheat (Triticum aestivum L.). Front. Plant Sci. 6:770. 10.3389/fpls.2015.00770 26483801 PMC4588698

[B49] Qing D. J. Lu H. F. Li N. Dong H. T. Dong D. F. Li Y. Z. (2009). Comparative profiles of gene expression in leaves and roots of maize seedlings under conditions of salt stress and the removal of salt stress. Plant Cell Physiol. 50, 889–903. 10.1093/pcp/pcp038 19264788

[B50] Quevillon E. Silventoinen V. Pillai S. Harte N. Mulder N. Apweiler R.. (2005). InterProScan: protein domains identifier. Nucleic Acids Res. 33, W116–W120. 10.1093/nar/gki442 15980438 PMC1160203

[B51] Rezaei M. K. Shobbar Z. S. Shahbazi M. Abedini R. Zare S. (2013). Glutathione S-transferase (GST) family in barley: identification of members, enzyme activity, and gene expression pattern. J. Plant. Physiol. 170, 1277–1284. 10.1016/j.jplph.2013.04.005 23664583

[B52] Saeed A. I. Sharov V. White J. Li J. Liang W. Bhagabati N.. (2003). TM4: A free, open-source system for microarray data management and analysis. Biotechniques 34, 374–378. 12613259 10.2144/03342mt01

[B53] Sappl P. G. Carroll A. J. Clifton R. Lister R. Whelan J. Harvey M. A.. (2009). The Arabidopsis glutathione transferase gene family displays complex stress regulation and co-silencing multiple genes results in altered metabolic sensitivity to oxidative stress. Plant J. 58, 53–68. 10.1111/j.1365-313X.2008.03761.x 19067976

[B54] Scarponi L. Quagliarini E. Buono D. D. (2006). Induction of wheat and maize glutathione S-transferase by some herbicide safeners and their effect on enzyme activity against butachlor and terbuthylazine. Pest Manag. Sci. 62, 927–932. 10.1002/ps.1258 16835885

[B55] Shabala S. (2013). Learning from halophytes: physiological basis and strategies to improve abiotic stress tolerance in crops. Ann. Bot. 112, 1209–1221. 10.1093/aob/mct205 24085482 PMC3806534

[B56] Sharma R. Sahoo A. Devendran R. Jain M. (2014). Over-expression of a rice tau class glutathione s-transferase gene improves tolerance to salinity and oxidative stresses in Arabidopsis. PLoS ONE 9:e92900. 10.1371/journal.pone.0092900 24663444 PMC3963979

[B57] Sheehan D. Meade G. Foley V. M. Dowd C. A. (2001). Structure, function and evolution of glutathione transferases: implications for classification of non-mammalian members of an ancient enzyme superfamily. Biochem. J. 360, 1–16. 10.1042/bj3600001 11695986 PMC1222196

[B58] Skopelitou K. Muleta A. W. Pavli O. Skaracis G. N. Flemetakis G. N. Papageorgiou A. C.. (2012). Overlapping protective roles for glutathione transferase gene family members in chemical and oxidative stress response in *Agrobacterium tumefaciens*. Funct. Integr. Genomics 12, 157–172. 10.1007/s10142-011-0248-x 21909786

[B59] Soranzo N. Gorla M. S. Mizzi L. Toma G. D. Frova C. (2004). Organisation and structural evolution of the rice glutathione S-transferase gene family. Mol. Genet. Genomics 271, 511–521. 10.1007/s00438-004-1006-8 15069639

[B60] Tamura K. Peterson D. Peterson N. Stecher G. Nei M. Kumar S. (2011). MEGA5: molecular evolutionary genetics analysis using maximum likelihood, evolutionary distance, and maximum parsimony methods. Mol. Biol. Evol. 28, 2731–2739. 10.1093/molbev/msr121 21546353 PMC3203626

[B61] Tanaka K. M. Takahasi K. R. Takano-Shimizu T. (2009). Enhanced fixation and preservation of a newly arisen duplicate gene by masking deleterious loss-of-function mutations. Genet. Res. (Camb) 91, 267–280. 10.1017/S0016672309000196 19640322

[B62] Thom R. Dixon D. P. Edwards R. Cole D. J. Lapthorn A. J. (2001). The structure of a zeta class glutathione S-transferase from arabidopsis thaliana: characterisation of a gst with novel active-site architecture and a putative role in tyrosine catabolism. J. Mol. Biol. 308, 949–962. 10.1006/jmbi.2001.4638 11352584

[B63] Urano J. Nakagawa T. Maki Y. Masumura T. Tanaka K. Murata N.. (2000). Molecular cloning and characterization of a rice dehydroascorbate reductase. FEBS Lett. 466, 107–111. 10.1016/S0014-5793(99)01768-8 10648822

[B64] Wagner U. Edwards R. Dixon D. P. Mauch F. (2002). Probing the diversity of the arabidopsis glutathione S-transferase gene family. Plant Mol. Biol. 49, 515–532. 10.1023/A:1015557300450 12090627

[B65] Wang K. Wang Z. Li F. Ye W. Wang J. Song G.. (2012). The draft genome of a diploid cotton Gossypium raimondii. Nat. Genet. 44, 1098–1103. 10.1038/ng.2371 22922876

[B66] Xu G. Guo C. Shan H. Kong H. (2012). Divergence of duplicate genes in exon-intron structure. Proc. Natl. Acad. Sci. U.S.A. 109, 1187–1192. 10.1073/pnas.1109047109 22232673 PMC3268293

[B67] Yang Q. Liu Y. J. Zeng Q. Y. (2014). Biochemical functions of the glutathione transferase supergene family of Larix kaempferi. Plant Physiol. Biochem. 77, 99–107. 10.1016/j.plaphy.2014.02.003 24583343

[B68] Yu C. S. Lin C. J. Hwang J. K. (2004). Predicting subcellular localization of proteins for Gram-negative bacteria by support vector machines based on n-peptide compositions. Protein Sci. 13, 1402–1406. 10.1110/ps.03479604 15096640 PMC2286765

[B69] Zhang J. (2003). Evolution by gene duplication: an update. Trends Ecol. Evol. (Amst). 18, 292–298. 10.1016/S0169-5347(03)00033-8

[B70] Zhao Y. Cai M. Zhang X. Li Y. Zhang J. Zhang H.. (2014). Genome-wide identification, evolution and expression analysis of mTERF gene family in maize. PLoS ONE 9:e94126. 10.1371/journal.pone.0094126 24718683 PMC3981765

[B71] Zhou M. L. Yang X. B. Zhang Q. Zhou M. Zhao E. Z. Tang Y. X.. (2013). Induction of annexin by heavy metals and jasmonic acid in Zea mays. Funct. Integr. Genomics 13, 241–251. 10.1007/s10142-013-0316-5 23474989

[B72] Zhu J. K. (2002). Salt and drought stress signal transduction in plants. Annu. Rev. Plant Biol. 53, 247–273. 10.1146/annurev.arplant.53.091401.143329 12221975 PMC3128348

