# Supplementary figures and images for: Glutathione S-Transferase Gene Family in Gossypium raimondii and G. arboreum: Comparative Genomic Study and their Expression under Salt Stress
Source: Front Plant Sci. 2016 Feb 12;7:139. doi: 10.3389/fpls.2016.00139 (PMC4751282; doi:10.3389/fpls.2016.00139)

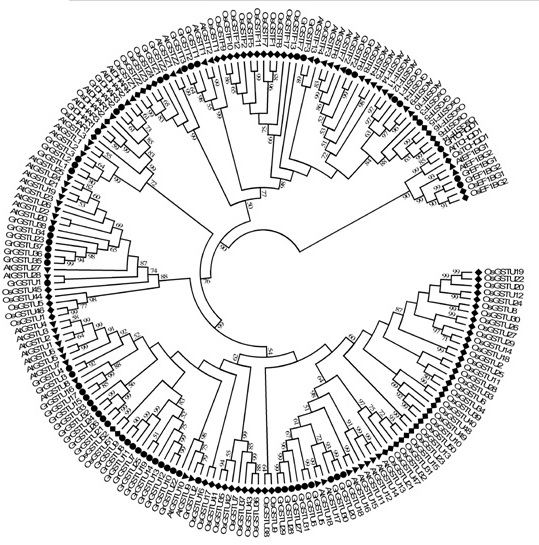

Supplement: Supplementary Figure 1 — Phylogenetic analysis of GST genes from G. raimondii, Arabidopsis, and rice. The unrooted phylogentic tree was constructed using MEGA 5.2 by Minimum Evolution method and the bootstrap test was performed with 1000 replicates. Percentage bootstrap scores of >50% were displayed. The GST genes from G. raimondii, Arabidopsis and rice were marked with the dots, triangles, and rhombuses, respectively. [file Image1.JPEG]

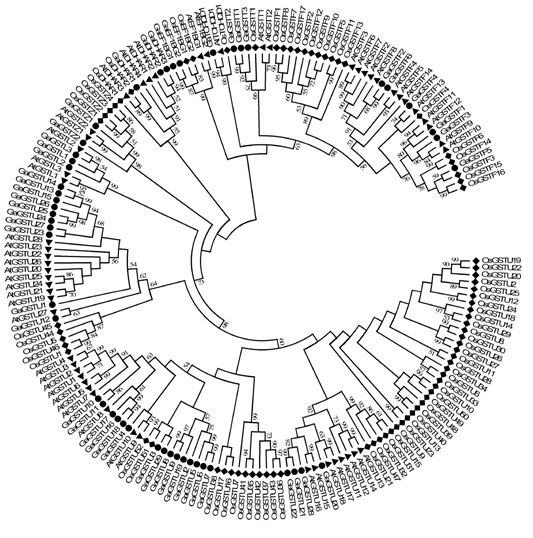

Supplement: Supplementary Figure 2 — Phylogenetic analysis of GST genes from G. arboreum, Arabidopsis, and rice. The unrooted phylogentic tree was constructed using MEGA 5.2 by Minimum Evolution method and the bootstrap test was performed with 1000 replicates. Percentage bootstrap scores of >50% were displayed. The GST genes from G. arboreum, Arabidopsis and rice were marked with the dots, triangles, and rhombuses, respectively. [file Image2.JPEG]

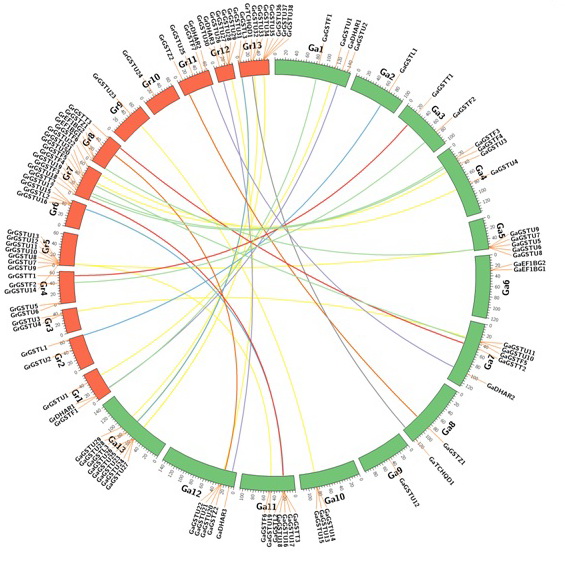

Supplement: Supplementary Figure 3 — Locations and orthologs of GST genes in G. raimondii and G. arboreum. The picture was generated by Circos software. The chromosomes of G. raimondii and G. arboreum were shown with different colors and labeled as Gr and Ga, respectively. The putative orthologous genes belonging to the Tau, Phi, Lambda, DHAR, Zeta, Theta, and TCHQD1 subfamilies were connected by yellow, green, blue, purple, orange, red, and gray lines, respectively. [file Image3.JPEG]
