# Supplementary material for: Glutathione S-Transferase Gene Family in Gossypium raimondii and G. arboreum: Comparative Genomic Study and their Expression under Salt Stress
Source: Front Plant Sci. 2016 Feb 12;7:139. doi: 10.3389/fpls.2016.00139 (PMC4751282; doi:10.3389/fpls.2016.00139)
Supplement: Supplementary Table 1 — PCR primers used in this study. [file Table1.DOC]

**Supplementary Table 1. PCR primers used in this study.**

| Gene name | Forward primer (5'-3') | Reverse primer (5'-3') |
| --- | --- | --- |
| *GrGSTU2* | CCCACTGCTACACTCTTGGG | ATGTGGTTGGGAGCCATTGT |
| *GrGSTU13* | CTTGGCTTAGTCCGTACGTTT | CGGTTTGCCGTCGTGAA |
| *GrGSTU16* | GGCAAGCCCTTACAGCTACA | GCAAATCGCTCTTGTTGCCA |
| *GrGSTU28* | AGTGTTGTTTGGTGCAACGG | CTCCAACAGCACCATCCCTT |
| *GrGSTU29* | CATGAGGCGGTGAAGGATGT | TAGCTTTCAAGACCCTCGCC |
| *GrGSTU30* | AAAATGGGCGGACAGGTTCT | CGCCAGCGGCTCTTATTTTG |
| *GrGSTU33* | GGTGTTGGGGACAAAGCAGA | GAACTGGGTTCAGTGCCAGA |
| *GrGSTU35* | GGAAAAGGAGCTTGGGGACA | AACGTTTCTTCATCTCCAGGAT |
| *GrGSTU38* | GGGGGACAAAACATTGGGGTA | CACCCTGCCTCTATGCTGAAA |
| *GrDHAR1* | AGCATTGGTTGGCTCGAAGA | CGCTAGAGATAAGTCCGCCG |
| *GrDHAR2* | TTCGGTCGGGAATCTTTCGT | CATTGACCTTCGGTGCCCAT |
| *GrGSTZ1* | GGTTGCCAGCATAGTCACCT | CTCCGGTTGCATATTTGCCG |
| *GrGSTZ2* | TCTGGCTGGGATTGAACGATT | ATCCGGCTGATTCTCTGGCT |
| *GrTCHQD1* | CTCGATTGGTGCTTCTGGGT | ACCAAACCCCAGTAGTCTGC |
| *GrGSTF2* | AACTCTTGTTGCCGGGTTCT | GTGTCGGTGGTCATCCCAAA |
| *GrGSTF4* | GGTGTATGGCCCAGCCTTT | TCCACTTGACCCCTTTCCTC |
| *GrGSTF5* | AGCCCTTTGGTCAAGTACCG | TACCAACAAATCGCCCGTGA |
| *GrGSTF7* | TGGCCAGATAGCGGCATTAG | GCGAATGAGTGCTTGGTTGG |
| *GrEF1BG1* | GCCGAACCAAAGAAAGAAGC | GGAGGCAGCAAATCAAGAGG |
| *GaGSTU3* | ATCGGCCTGGATGCAAGAAT | TCATCTTGTCACGAGGAGGC |
| *GaGSTU7* | TGTTGGCGCCTATGGAAGAG | CAGGGTGTTCGCTGAAATGC |
| *GaGSTU8* | ACATGCGTTGATCGGAGTGA | TCTTCGAAAACCCCAAGCCA |
| *GaGSTU15* | GCATTGAAACAGAGTGCCCC | TTGATCAGGCAGGGACTTGG |
| *GaGSTU16* | GCCAAGGCTCGATTCTGGAT | CTCTCTCTCCTCCTCAGGGC |
| *GaGSTU19* | GTCCACTGCTTCTCCAGCAT | GTTGGCAGCAGGGGATTTTG |
| *GaGSTU14* | CTGGGCGACAAAGGGAGAAG | AGCCTTGTTTCCAAGCCCTC |
| *GaGSTU20* | GGGATGGCTTAGAGTGACGG | AGAACCTGTCCGCCCATTTT |
| *GaGSTU24* | GCTCCTTTGCTCCCTTCTGA | ACTCTGCTCCCCAGGTCATA |
| *GaGSTU29* | TTGGGGACAAAGCAGAGCAT | GGATTCGGCAATGGGCTTTC |
| *GaDHAR1* | AGGGAAGGTTCCGGTAGTGA | ACCAATGCTTTTTCCTGCGG |
| *GaGSTZ1* | CTGGTGGACGGTGACATGAT | GCAGCCTGGAAATTGAGTGC |
| *GaTCHQD1* | GAAAGTGGTGATCGCTCGGA | TCTCCTCGGTCTCGTATGCT |
| *GaGSTF1* | ATGGGCAAGCAGGGTGATAC | AGCGTTGCTCGTAGATGTCC |
| *GaGSTF2* | AACTCTTGTTGCCGGGTTCT | GGAGGGTCGAACTGATGAGC |
| *GaGSTF3* | TGCTTCCAAGATGGGGTTCC | CAGGTCAGCAAGGCTGAAGA |
| *GaGSTF5* | GGCAGAAGGGCAGAGCTTTA | CACCTCCAGCACTTTAGCCA |
| *GaGSTF6* | ACGTGTACGAGGCTCGATTG | AAGCACAAACATGGGGACGA |
| *GaGSTT1* | GCTTGGCCATCCATCGAATC | GCCCATCACCCTTCAACCAA |
| *GaGSTT3* | GCTTGGCCGTCCATTGAATC | CCTTGAGCCAAACGGACTCT |
| *GaGSTL2* | GCAGAGCAACCCGCTATCTT | AGGACGGCACCTTGTTTTCA |
| *UBQ7* | GAAGGCATTCCACCTGACCAAC | CTTGACCTTCTTCTTCTTGTGCTTG |
