# Supplementary material for: Glutathione S-Transferase Gene Family in Gossypium raimondii and G. arboreum: Comparative Genomic Study and their Expression under Salt Stress
Source: Front Plant Sci. 2016 Feb 12;7:139. doi: 10.3389/fpls.2016.00139 (PMC4751282; doi:10.3389/fpls.2016.00139)
Supplement: Supplementary Table 2 — The information of partial GST genes and mPGES2 and C_pmega_lke subfamilies in G. raimondii and G. arboreum. [file Table2.DOC]

| **Supplementary Table 2**. **The information of partial *GST* genes and mPGES2 and C_pmega_lke subfamilies in G. raimondii and G. arboreum.** | | | | | |
| --- | --- | --- | --- | --- | --- |
| Species | Gene identifier | Genomics position | CDS | Size (AA) | Domain/  subfamily  subfamily |
| *G. raimondii* | Gorai.001G236000.1 | Chr01: 47366934-47369290 | 411 | 136 | GST_C |
|  | Gorai.004G032100.1 | Chr04: 2626962-2628597 | 693 | 230 | GST_N_3 |
|  | Gorai.004G211200.1 | Chr04: 54411153-54414131 | 306 | 101 | GST_N |
|  | Gorai.005G037600.1 | Chr05: 3540356 3540757 | 402 | 133 | GST_C |
|  | Gorai.005G038300.1 | Chr05: 3601006-3602898 | 273 | 90 | GST_C |
|  | Gorai.005G038400.1 | Chr05: 3609845-3610416 | 396 | 131 | GST_N |
|  | Gorai.005G125800.1 | Chr05: 28094887-28095674 | 357 | 118 | GST_C |
|  | Gorai.007G023200.1 | Chr07: 1660852-1661620 | 372 | 123 | GST_C_2 |
|  | Gorai.008G063900.1 | Chr08: 10242665-10245139 | 297 | 98 | GST_C |
|  | Gorai.008G246200.1 | Chr08: 53065682-53067270 | 270 | 89 | GST_N |
|  | Gorai.009G042300.1 | Chr09: 3090356-3095847 | 297 | 98 | GST_C |
|  | Gorai.009G265800.1 | Chr09: 22025683-22033076 | 1047 | 348 | GST_N_3 |
|  | Gorai.010G095500.1 | Chr10: 15844304 15845279 | 420 | 139 | GST_C |
|  | Gorai.011G207100.1 | Chr11: 49878965-49879528 | 417 | 138 | GST_C |
|  | Gorai.012G108800.1 | Chr12: 24468775-24469673 | 453 | 150 | GST_C |
|  | Gorai.013G153600.1 | Chr13: 42214444-42215266 | 486 | 161 | GST_C |
|  | Gorai.005G133000.1 | Chr05: 33229869-33235620 | 990 | 329 | mPGES2 |
|  | Gorai.009G193000.1 | Chr09: 14835025-14838359 | 969 | 322 | mPGES2 |
|  | Gorai.001G181000.1 | Chr01: 27687595-27689583 | 1220 | 406 | C_omega_like |
|  | Gorai.009G151800.1 | Chr09: 11542542-11545434 | 1209 | 402 | C_omega_like |
| *G. arboreum* | Cotton_A_25982 | Chr03: 24935842-24937002 | 450 | 149 | GST_C |
|  | Cotton_A_25154 | Chr04: 75545245-75559637 | 1425 | 474 | GST_N |
|  | Cotton_A_24524 | Chr05: 56798570-56798860 | 291 | 96 | GST_C |
|  | Cotton_A_35325 | Chr07: 85205377-85205721 | 345 | 114 | GST_C |
|  | Cotton_A_22221 | Chr09: 59639579-59639863 | 285 | 94 | GST_C |
|  | Cotton_A_35373 | Chr08: 33328418-33328657 | 240 | 79 | GST_N |
|  | Cotton_A_20279 | Chr12: 67623898-67627176 | 2184 | 727 | GST_C |
|  | Cotton_A_24702 | Chr09: 293537-297509 | 2187 | 728 | GST_C |
|  | Cotton_A_04567 | Chr06: 104463358-104470469 | 1005 | 335 | GST_N |
|  | Cotton_A_22666 | Chr12: 87084378-87084700 | 249 | 82 | GST_C |
|  | Cotton_A_22664 | chr12: 87089956-87090366 | 411 | 136 | GST_C |
|  | Cotton_A_22663 | Chr12: 87090769-87091627 | 351 | 116 | GST_C |
|  | Cotton_A_28313 | Chr01: 30400551-30402218 | 1131 | 376 | C_Omega_like |
|  | Cotton_A_26148 | Chr10: 16093480-16094983 | 978 | 325 | C_Omega_like |
|  | Cotton_A_16442 | Chr08: 83927326-83930308 | 966 | 321 | mPGES2 |
