# Supplementary material for: Glutathione S-Transferase Gene Family in Gossypium raimondii and G. arboreum: Comparative Genomic Study and their Expression under Salt Stress
Source: Front Plant Sci. 2016 Feb 12;7:139. doi: 10.3389/fpls.2016.00139 (PMC4751282; doi:10.3389/fpls.2016.00139)
Supplement: Supplementary Table 3 — The cis-elements involved in salt stress response in the promoter regions of GrGSTs and GaGSTs. [file Table3.DOC]

**Supplementary Table 3. The *cis*-elements involved in salt stress response in the promoter regions of *GrGSTs* and *GaGSTs*. _reprensets no DRE.**

| Gene name | Drought Responsive Elements (DRE) | | | Gene name | Drought Responsive Elements (DRE) | | |
| --- | --- | --- | --- | --- | --- | --- | --- |
|  | S000401 | S000402 | S000418 |  | S000401 | S000402 | S000418 |
| *GrGSTU1* | _ | _ | _ | *GrEF1Bγ2* | _ | _ | _ |
| *GrGSTU2* | 1 | 1 | 2 | *GrDHAR1* | 1 | 1 | 1 |
| *GrGSTU3* | _ | _ | _ | *GrDHAR2* | 1 | _ | _ |
| *GrGSTU4* | _ | _ | _ | *GrDHAR3* | _ | _ | _ |
| *GrGSTU5* | _ | _ | _ | *GrTCHQD1* | 1 | _ | _ |
| *GrGSTU6* | _ | _ | _ | *GaGSTU1* | _ | _ | _ |
| *GrGSTU7* | _ | _ | _ | *GaGSTU2* | _ | _ | _ |
| *GrGSTU8* | _ | _ | _ | *GaGSTU3* | 1 | 2 | _ |
| *GrGSTU9* | _ | _ | _ | *GaGSTU4* | _ | _ | _ |
| *GrGSTU10* | _ | _ | _ | *GaGSTU5* | _ | _ | _ |
| *GrGSTU11* | _ | _ | _ | *GaGSTU6* | _ | _ | _ |
| *GrGSTU12* | _ | _ | _ | *GaGSTU7* | 1 | 1 | _ |
| *GrGSTU13* | _ | 1 | 2 | *GaGSTU8* | 1 | 1 | _ |
| *GrGSTU14* | _ | _ | _ | *GaGSTU9* | _ | _ | _ |
| *GrGSTU15* | _ | _ | _ | *GaGSTU10* | _ | _ | _ |
| *GrGSTU16* | _ | _ | 1 | *GaGSTU11* | _ | _ | _ |
| *GrGSTU17* | _ | _ | _ | *GaGSTU12* | _ | _ | _ |
| *GrGSTU18* | _ | 1 | 1 | *GaGSTU13* | _ | _ | _ |
| *GrGSTU19* | _ | _ | _ | *GaGSTU14* | 1 | 1 | _ |
| *GrGSTU20* | _ | _ | _ | *GaGSTU15* | _ | 1 | _ |
| *GrGSTU21* | _ | _ | _ | *GaGSTU16* | 1 | 1 | _ |
| *GrGSTU22* | _ | _ | _ | *GaGSTU17* | _ | _ | _ |
| *GrGSTU23* | _ | _ | _ | *GaGSTU18* | _ | _ | _ |
| *GrGSTU24* | _ | _ | _ | *GaGSTU19* | 3 | 4 | _ |
| *GrGSTU25* | _ | _ | _ | *GaGSTU20* | 2 | 2 | _ |
| *GrGSTU26* | _ | _ | _ | *GaGSTU21* | _ | _ | _ |
| *GrGSTU27* | _ | _ | _ | *GaGSTU22* | _ | _ | _ |
| *GrGSTU28* | _ | 1 | 1 | *GaGSTU23* | _ | _ | _ |
| *GrGSTU29* | _ | 8 | 8 | *GaGSTU24* | 3 | 4 | _ |
| *GrGSTU30* | 1 | 1 | 1 | *GaGSTU25* | _ | _ | _ |
| *GrGSTU31* | _ | _ | _ | *GaGSTU26* | _ | _ | _ |
| *GrGSTU32* | _ | _ | _ | *GaGSTU27* | _ | _ | _ |
| *GrGSTU33* | _ | 1 | 1 | *GaGSTU28* | _ | _ | _ |
| *GrGSTU34* | _ | _ | _ | *GaGSTU29* | 1 | 1 | _ |
| *GrGSTU35* | _ | 1 | 1 | *GaGSTF1* | 1 | 1 | _ |
| *GrGSTU36* | _ | _ | _ | *GaGSTF2* | _ | _ | 1 |
| *GrGSTU37* | _ | _ | _ | *GaGSTF3* | 1 | 2 | _ |
| *GrGSTU38* | _ | _ | 1 | *GaGSTF4* | _ | _ | _ |
| *GrGSTF1* | _ | _ | _ | *GaGSTF5* | 1 | 1 | _ |
| **Supplementary Table 3.** (continued) | | | | | | | |
| *GrGSTF2* | 2 | _ | _ | *GaGSTF6* | 1 | 1 | _ |
| *GrGSTF3* | _ | _ | _ | *GaGSTT1* | _ | 1 | _ |
| *GrGSTF4* | _ | _ | 1 | *GaGSTT2* | _ | _ | _ |
| *GrGSTF5* | _ | 1 | 1 | *GaGSTT3* | _ | 1 | _ |
| *GrGSTF6* | _ | _ | _ | *GaGSTZ1* | _ | 1 | _ |
| *GrGSTF7* | 1 | _ | 1 | *GaGSTZ2* | _ | _ | _ |
| *GrGSTT1* | _ | _ | _ | *GaGSTL1* | _ | _ | _ |
| *GrGSTT2* | _ | _ | _ | *GaGSTL2* | _ | 2 | _ |
| *GrGSTT3* | _ | _ | _ | *GaGSTL3* | _ | _ | _ |
| *GrGSTZ1* | _ | _ | 1 | *GaEF1Bγ1* | _ | _ | _ |
| *GrGSTZ2* | _ | 2 | 2 | *GaEF1Bγ2* | _ | _ | _ |
| *GrGSTL1* | _ | _ | _ | *GaDHAR1* | 1 | 1 | _ |
| *GrGSTL2* | _ | _ | _ | *GaDHAR2* | _ | _ | _ |
| *GrGSTL3* | _ | _ | _ | *GaDHAR3* | _ | _ | _ |
| *GrEF1Bγ1* | _ | _ | _ | *GaTCHQD1* | 1 | 1 | 1 |
